# Shaping future forests: how can ecophysiology support climate‐smart forest management?

**DOI:** 10.1111/nph.71007

**Published:** 2026-02-23

**Authors:** Arthur Gessler, José M. Grünzweig, Laura Bigio, Henrik Hartmann, Nate McDowell, Frank Krumm, Arun K. Bose, Andreas Rigling, Harald Bugmann, Valentina Vitali, Pascal Schneider, J. Jelle Lever, Janine Schweier, Anne Kempel, Niklaus E. Zimmermann, Philipp Brun, Jürgen Bauhus, Micah Wilhelm, Alessandra Bottero

**Affiliations:** ^1^ Swiss Federal Research Institute WSL Birmensdorf 8903 Switzerland; ^2^ Institute for Terrestrial Ecosystems ETH Zurich Zurich 8092 Switzerland; ^3^ The Robert H. Smith Faculty of Agriculture, Food and Environment The Hebrew University of Jerusalem Rehovot 76100 Israel; ^4^ Julius Kühn‐Institute for Forest Protection Quedlinburg 06484 Germany; ^5^ Faculty of Forest Sciences and Forest Ecology Georg‐August‐University Göttingen Göttingen 37077 Germany; ^6^ Department of Biogeochemical Processes Max Planck Institute for Biogeochemistry Jena 07745 Germany; ^7^ Atmospheric, Climate, and Earth Sciences Division Pacific Northwest National Laboratory Richland WA 99354 USA; ^8^ Climate Change, Extremes and Natural Hazards in Alpine Regions Research Centre CERC Davos 7260 Switzerland; ^9^ WSL Institute for Snow and Avalanche Research SLF Davos 7260 Switzerland; ^10^ Chair of Silviculture and Future Forests Cluster of Excellence University of Freiburg Freiburg 79085 Germany

**Keywords:** biodiversity, climate change, ecophysiology, ecosystem services, forestry, pathogens, production time, stand density

## Abstract

Climate change, particularly the associated increase in extreme events and disturbances, threatens the numerous environmental, social, and economic benefits that forests provide, both locally and globally. Heat and drought pose significant risks to forest ecosystems; the anticipated future climate is expected to exacerbate this trend. Management interventions should aim to maximise the provision of ecological functions amid the uncertain conditions ahead. A better understanding of the mechanisms regulating forest responses to drought, heat, pests, and diseases – and how management interventions interact with these – is necessary for evidence‐based, climate‐smart forest management. We first provide an overview of the ecophysiological mechanisms that drive the loss of ecosystem functioning induced by heat and drought. We then explore how various commonly adopted management interventions at the stand level – such as tree species selection and mixture, stand density regulation, measures to optimise stand structure, tree height, and age distribution, as well as nutrient management – may positively or negatively influence forest ecophysiological responses to heat and drought. In this work, we present a mechanism‐based critical assessment of forest management practices to support climate‐smart forestry/forest management in response to shifting environmental and climatic conditions.

## সারাংশ

জলবায়ু পরিবর্তন, বিশেষ করে চরম ঘটনা ও ব্যাঘাতের বৃদ্ধির কারণে, স্থানীয় ও বৈশ্বিকভাবে বন যে অসংখ্য পরিবেশগত, সামাজিক ও অর্থনৈতিক সুবিধা প্রদান করে, তা হুমকির মুখে ফেলছে। তাপ ও খরা বন বাস্তুতন্ত্রের জন্য উল্লেখযোগ্য ঝুঁকি সৃষ্টি করে; প্রত্যাশিত ভবিষ্যৎ জলবায়ু এই প্রবণতাকে আরও তীব্র করবে। অনিশ্চিত পরিস্থিতিতে বাস্তুতাত্ত্বিক কার্যকারিতা সর্বাধিক করার লক্ষ্যে বন ব্যবস্থাপনায় হস্তক্ষেপ করা উচিত। তাপ, খরা, কীটপতঙ্গ ও রোগ নিয়ন্ত্রণকারী প্রক্রিয়াগুলি এবং ব্যবস্থাপনা হস্তক্ষেপগুলি কীভাবে এগুলির সাথে মিথস্ক্রিয়া করে তা আরও ভালোভাবে বোঝা, জলবায়ু‐স্মার্ট বন ব্যবস্থাপনার জন্য অপরিহার্য। প্রথমে আমরা তাপ ও খরার কারণে বাস্তুতন্ত্রের কার্যকারিতা ক্ষয়ের পেছনে কাজ করা বাস্তুতন্ত্র‐ভিত্তিক প্রক্রিয়াগুলির একটি সারসংক্ষেপ প্রদান করেছি। এরপর আমরা অন্বেষণ করি কীভাবে স্ট্যান্ড স্তরে সাধারণভাবে গৃহীত বিভিন্ন ব্যবস্থাপনা হস্তক্ষেপ ‐ যেমন গাছের প্রজাতি নির্বাচন ও মিশ্রণ, স্ট্যান্ড ঘনত্ব নিয়ন্ত্রণ, গাছের উচ্চতা ও বয়সের বণ্টন, এবং পুষ্টি ব্যবস্থাপনা ‐ তাপ ও খরার প্রতি বন‐বাস্তুতন্ত্রের ইকোফিজিওলজিক্যাল প্রতিক্রিয়াকে ইতিবাচক বা নেতিবাচকভাবে প্রভাবিত করতে পারে। এই কাজে, আমরা পরিবর্তনশীল পরিবেশগত ও জলবায়ুগত অবস্থার প্রতিক্রিয়ায় জলবায়ু‐স্মার্ট বনসম্পদ/বন ব্যবস্থাপনাকে সমর্থন করার জন্য বন ব্যবস্থাপনার অনুশীলনগুলির একটি যান্ত্রিক‐ভিত্তিক সমালোচনামূলক মূল্যায়ন উপস্থাপন করছি।

## Samenvatting

Klimaatverandering, en de daarmee gepaard gaande toename van extreme weersomstandigheden, vormt een ernstige bedreiging voor de ecologische, sociale en economische functies van bossen, zowel lokaal als wereldwijd. Vooral hitte en droogte brengen grote risico's met zich mee voor bosecosystemen, en deze druk zal onder toekomstige klimaatomstandigheden verder toenemen. Bosbeheer moet daarom gericht zijn op het behoud en de versterking van ecosysteemfuncties en ‐diensten, ondanks de onzekerheden van de toekomst. Een beter begrip van de mechanismen die de reacties van bossen op hitte, droogte, plagen en ziekten bepalen, en van de wisselwerking met beheermaatregelen, is essentieel voor evidence‐based en klimaatslim bosbeheer. In dit werk beschrijven we eerst de ecologisch‐fysiologische processen die ten grondslag liggen aan functieverlies door hitte en droogte. Vervolgens analyseren we hoe gangbare beheermaatregelen op bestandsniveau, zoals boomsoortenselectie en ‐menging, regulering van bestandsdichtheid, structuur, leeftijdsverdeling en nutriëntenbeheer, deze reacties kunnen beïnvloeden. Tot slot geven we een kritische, mechanistische evaluatie van bosbeheerpraktijken met het oog op klimaatslim bosbeheer onder veranderende milieuomstandigheden.

## Résumé

Le changement climatique, avec l’augmentation des événements extrêmes et des perturbations, met en péril les nombreux avantages environnementaux, sociaux et économiques que les forêts procurent, aussi bien localement que sur le plan mondial. Il ne fait aucun doute que la chaleur et la sécheresse représentent des dangers majeurs pour les écosystèmes forestiers. Les prévisions climatiques sont sans équivoque: cette tendance va clairement s’aggraver. Les interventions de gestion doivent absolument viser à maximiser la fourniture de fonctions écologiques dans les conditions incertaines à venir. Nous avons aujourd’hui les outils pour comprendre et maîtriser les réponses des forêts face aux défis du climat. En saisissant avec précision les mécanismes régissant la sécheresse, la chaleur, les ravageurs et les maladies, nous pouvons intervenir de manière stratégique et efficace. Une gestion forestière à la fois intelligente et fondée sur des preuves, pour un avenir durable et résilient. Nous commencerons par vous fournir un aperçu des mécanismes écophysiologiques qui provoquent la perte de fonctionnement des écosystèmes. Cette perte est causée par la chaleur et la sécheresse. Nous avons ensuite exploré comment diverses interventions de gestion communément adoptées au niveau du peuplement – telles que la sélection et le mélange d’espèces d’arbres, la régulation de la densité du peuplement, les mesures pour optimiser la structure du peuplement, la distribution de la hauteur et de l’âge des arbres, ainsi que la gestion des nutriments – peuvent influencer positivement ou négativement les réponses écophysiologiques forestières à la chaleur et à la sécheresse. Dans cette étude, nous dévoilons une analyse approfondie et éclairée, fondée sur les mécanismes des pratiques de gestion forestière. Notre objectif: soutenir la foresterie et la gestion forestière intelligentes face au climat, en réponse aux conditions environnementales et climatiques en constante évolution.

## Zusammenfassung

Der Klimawandel, insbesondere die damit verbundene Zunahme extremer Ereignisse und Störungen, bedroht die zahlreichen ökologischen, sozialen und wirtschaftlichen Leistungen, die Wälder sowohl lokal als auch global bieten. Hitze und Dürre stellen erhebliche Risiken für Waldökosysteme dar; das erwartete zukünftige Klima dürfte diesen Trend noch verstärken. Managementmaßnahmen sollten darauf abzielen, die verschiedenen Funktionen und Leistungen unter den unsicheren Bedingungen der Zukunft zu maximieren. Ein besseres Verständnis der Mechanismen, die die Reaktionen der Wälder auf Dürre, Hitze, Schädlinge und Krankheiten regulieren – und wie Managementmaßnahmen mit diesen interagieren – ist für eine evidenzbasierte, «Klima‐smarte» Waldbewirtschaftung erforderlich. Zunächst geben wir einen Überblick über die ökophysiologischen Mechanismen, die den durch Hitze und Dürre verursachten Verlust der Ökosystemfunktionen vorantreiben. Anschließend untersuchen wir, wie verschiedene gängige Bewirtschaftungsmaßnahmen auf Bestandesebene – etwa die Auswahl und Mischung von Baumarten, die Regulierung der Bestandsdichte, Maßnahmen zur Optimierung der Bestandsstruktur, der Baumhöhe und der Altersverteilung sowie das Nährstoffmanagement – die ökophysiologischen Reaktionen des Waldes auf Hitze und Trockenheit positiv oder negativ beeinflussen können. In dieser Arbeit präsentieren wir eine mechanismenbasierte kritische Bewertung von Waldbewirtschaftungspraktiken zur Unterstützung einer klimafreundlichen Forstwirtschaft/Waldbewirtschaftung, die auf sich verändernde Umwelt‐ und Klimabedingungen reagieren kann.

## Riepilogo

Il cambiamento climatico, in particolare il conseguente aumento degli eventi estremi e dei disturbi, minaccia i numerosi benefici ambientali, sociali ed economici che le foreste forniscono, sia a livello locale sia globale. Il caldo e la siccità rappresentano rischi significativi per gli ecosistemi forestali; il clima futuro previsto è destinato ad accentuare questa tendenza. Gli interventi di gestione forestale dovrebbero mirare a massimizzare la fornitura di funzioni ecologiche in condizioni di elevata incertezza. Una migliore comprensione dei meccanismi che regolano le risposte delle foreste a siccità, caldo, patogeni e malattie – e di come gli interventi gestionali interagiscano con tali meccanismi — è necessaria per una gestione forestale basata su evidenze e climaticamente intelligente. In primo luogo forniamo una panoramica dei meccanismi ecofisiologici che guidano la perdita di funzionalità degli ecosistemi forestali indotta da caldo e siccità. Successivamente analizziamo come diversi interventi gestionali comunemente adottati a livello di popolamento forestale – quali la selezione e la mescolanza delle specie arboree, la regolazione della densità del popolamento, le misure per ottimizzare la struttura del popolamento, l’altezza degli alberi e la distribuzione delle classi di età, nonché la gestione dei nutrienti – possano influenzare positivamente o negativamente le risposte ecofisiologiche delle foreste al caldo e alla siccità. In questo lavoro presentiamo una valutazione critica, basata sui meccanismi, delle pratiche di gestione forestale a supporto di una selvicoltura/gestione forestale climaticamente intelligente in risposta al cambiamento delle condizioni ambientali e climatiche.

**Table:** 

	Content	
	Summary	2778
I.	Introduction	2779
II.	Climate change and its impact on the ecophysiology of trees and forests	2782
III.	Climate‐smart management practices and their ecophysiology‐based impacts on forest resistance and resilience to climate change	2784
IV.	Dryland mechanisms as targets of CSF management practices	2803
V.	Conclusions	2805
	Acknowledgements	2805
	References	2805

## I. Introduction

Forests cover *c*. 30% of the global land area (FAO & UNEP, 2020), and provide numerous ecological functions and ecosystem services. Approximately 80% of the known terrestrial species world‐wide directly depend on forests and temporarily or permanently inhabit forest areas (FAO & UNEP, 2020), making these ecosystems crucial for biodiversity conservation (Foley *et al*., 2005). Forests play a substantial role in climate protection because they store large amounts of carbon above‐ and belowground, strongly contributing to the current terrestrial carbon sink. Forest cover influences climate through its effects on the water cycle and the surface radiative balance, via albedo and surface roughness, with substantial impacts on local to global scales (Bonan, 2008). Forests provide multiple ecosystem services simultaneously that are important for human livelihood and welfare, such as timber as a construction material and fibres for bioenergy and the chemical industry (cf Richardson *et al*., 2006). Especially in mountain regions, they protect human settlements and infrastructure from gravitational natural hazards occurring on steep slopes, such as avalanches and rockfall (Casteller *et al*., 2018; Frank *et al*., 2021). Taken together, forests are essential for life on Earth, as reflected in several Sustainable Development Goals related to these ecosystems globally (see United Nations, Department of Economic and Social Affairs: https://sdgs.un.org/topics/forests).

Numerous forest functions are jeopardised by global change. The expected rise in temperatures, along with more frequent and severe droughts, poses a threat to forests that already exhibit several signs of increased vulnerability (Allen *et al*., 2010, 2015; Xu *et al*., 2024), and carbon, water, and nutrient relations are strongly affected by emerging forest dieback (Anderegg *et al*., 2016b). Extreme climatic events such as severe and extended droughts (Chen *et al*., 2025), in the context of global warming also known as ‘hotter droughts’ (Allen *et al*., 2015), alone or in combination with increasing natural abiotic and biotic disturbances (Seidl *et al*., 2017), are altering the functioning of forest ecosystems around the globe (McDowell *et al*., 2018). Moreover, the tree species and genetic composition of many forests are changing in response to climate change and will continue to do so, impacting and altering forest ecosystem services (Hanewinkel *et al*., 2013; Wessely *et al*., 2024; Chakraborty *et al*., 2024a).

As human societies rely on multiple forest ecosystem services, forests have been influenced by human activities and interventions for millennia (Gobet *et al*., 2003; Blondel, 2006; Lewis *et al*., 2015). Less than 20% of the world's forests are unmanaged, primarily in the tropics and in remote, inaccessible areas of the boreal zone in Canada and Russia (Watson *et al*., 2018). Large proportions of forested regions in the temperate, Mediterranean, and boreal zones of Europe and the United States have been systematically managed for centuries (FOREST EUROPE *et al*., 2011; Hengeveld *et al*., 2012; McGrath *et al*., 2015; Palik *et al*., 2020). Forest management addresses a variety of environmental, social, and economic objectives, each emphasising different ecosystem services. Balancing these often competing demands makes management decisions highly complex and multifaceted (Duncker *et al*., 2012). Globalisation and climate change further increase this complexity, as future climatic conditions and the emergence of new biotic agents may pose threats to forests that are difficult to anticipate (Thrippleton *et al*., 2023) and may jeopardise the sustainability of forest service provision.

At present, multiple management concepts such as Sustainable Forest Management (Siry *et al*., 2018), integrated forest management (Krumm *et al*., 2020), close‐ or closer‐to‐nature forest management (Brang *et al*., 2014; Krumm *et al*., 2023), and climate‐smart forestry (CSF; Jandl *et al*., 2018) refer back to the principles of von Carlowitz (1732) and support the aims of sustainable forest development (Schweier *et al*., 2019). Here, we focus on CSF (see Box 1) as it provides a concept that can help policymakers and practitioners develop forestry governance and management strategies for forest adaptation and climate change mitigation, while also considering biodiversity and balanced ecosystem services (Nabuurs *et al*., 2018; Bowditch *et al*., 2020; Weatherall *et al*., 2022). Here we use adaptation in two different senses: (1) adaptation in a management sense comprises measures to adjust a forest (ecosystem) to new boundary conditions; and (2) adaptation in an evolutionary sense constitutes an evolutionary process where populations of a given species genetically change over generations through natural selection. In the same context, acclimation (also referred to as phenotypic plasticity) means reversible shifts in phenotype in response to environmental pressure/disturbance, resulting from active and passive responses. Moreover, we focus on stand‐level management, as this is the standard operational scale at which silvicultural practices are applied. We are aware that management at regional, catchment, and even larger scales is essential for water management (Sun *et al*., 2023) and biosphere–climate feedback (Bonan, 2008) and that the temporal and spatial continuity of forest cover is a key planning goal at these scales. However, on these scales, multi‐actor governance of forest resources is required, involving complex interactions among state, private, and civil society stakeholders at various levels (Mwangi & Wardell, 2012). Transferring ecophysiological principles to such multi‐level systems is beyond the scope of this article.

Box 1 **Definition of climate‐smart forestry (CSF)**

CSF draws from the concept of climate smartness in agriculture (FAO, 2010), which is ‘an approach that helps to guide actions needed to transform and reorient agricultural systems to effectively support development and ensure food security in a changing climate’. CSF is generally defined as a management practice that primarily enhances the resistance and resilience of forests against climate change (Nabuurs *et al*., 2018; Bowditch *et al*., 2020; Weatherall *et al*., 2022). This resistance and resilience should also aid climate change mitigation by maintaining or improving ecosystem functions. Central targets are to enhance forests' carbon sequestration, along with timber production, which supports a bio‐based economy with a reduced CO_2_ footprint. Furthermore, CSF seeks to balance different ecosystem services and maintain the multifunctionality of forests.

Climate change creates persistent environmental uncertainty, with unknown timeframes for stabilisation that challenge traditional forest management. Given that trees live for decades or even centuries, management decisions made today must account for highly uncertain future conditions across multiple time horizons that may comprise tipping points in the climate system (e.g. Heubel *et al*., 2025). This necessitates management versatility and agility as core principles, treating forest adaptation as a dynamic, ongoing process rather than a fixed endpoint. Management strategies must therefore be designed for continuous adjustment, incorporating adaptive principles that allow for corrections as climate trajectories evolve.

**Fig. 1:**
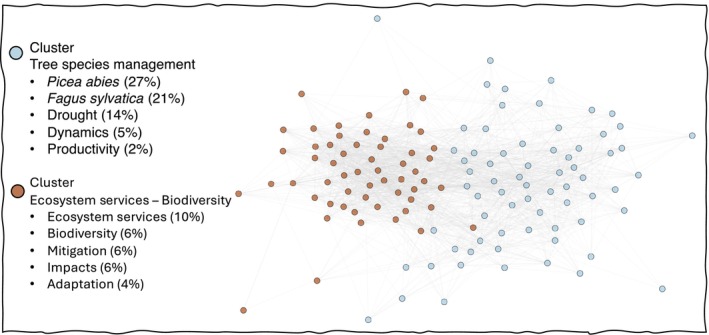
Results of the cluster analysis of papers published on climate‐smart forestry (CSF) using author keywords as connections between papers. Visualisation of publications retrieved from the Web of Science Core Collection using the search string: All fields: ‘climate smart forest*’ (*n* = 165). Two main clusters emerged: one focused on the management of major European tree species, and another on biodiversity and ecosystem functioning. The first cluster may indicate a bias towards Europe, where CSF and other management practices, such as integrated and close‐to‐nature forest management, were coined first. We applied the Newman clustering algorithm to partition the network of papers based on shared keywords (excluding search terms), grouping papers that were more strongly connected than expected by chance. Frequently shared keywords within each cluster are listed on the left, with percentages indicating their contribution to the cluster's network links. The distance between the nodes (i.e. papers) reflects the relative strength of interconnections, with peripheral papers positioned further from the centre. For methodological details, see Calamita *et al*. (2024).

Yet, it is largely untested to what extent CSF is founded on a comprehensive understanding of the underlying ecological processes. An examination of the Web of Science database yielded 165 papers on the topic of CSF, and they can be categorised into two primary clusters (Fig. 1). One cluster is primarily related to specific tree species management, emphasising two of the most important European tree species, *Picea abies* and *Fagus sylvatica*, the other to biodiversity and ecosystem service provision. The focus of the existing literature on specific species may be due to the fact that stands dominated by these two species have been shown to be susceptible to climate change (e.g. Schuldt *et al*., 2020), and CSF practices are applied to adapt existing stands. Based on the keywords provided by the authors within the clusters, mechanisms and processes are clearly not the primary focus of the publications, as ecophysiological and ecosystem process‐related keywords are not among those mentioned most frequently.

As the ongoing and – probably even more importantly – the expected future changes in climatic conditions exceed the range of empirical and experimental knowledge in forestry, solid scientific evidence on the processes and functioning of trees and forests under new boundary conditions is necessary to guide CSF. In this context, it is crucial to understand how heat, drought, and interactions with biotic factors influence the functioning of trees and forest ecosystems, particularly in terms of carbon balance and the relationships between water and nutrients (McDowell *et al*., 2011a; Gessler *et al*., 2017). In a second step, it is important to consider how management can mitigate and compensate for such impacts without significantly altering forest resistance and resilience, stressing the trees, or increasing the vulnerability of trees and forests to subsequent biotic and abiotic disturbances. A better mechanistic understanding of management impacts can ultimately improve process‐based models to project forest functioning under various climate and management scenarios (Fontes *et al*., 2010).

Recently, Grünzweig *et al*. (2022) have argued that ecosystems exposed to regular dry conditions are characterised by a typical set of traits that functionally convey so‐called ‘dryland mechanisms’ (cf Box 2). They also suggested that such mechanisms might, under climate change, start to play a more critical role in historically wetter climatic zones and could, thus, become crucial for maintaining ecosystem function. Hence, CSF could target these dryland mechanisms to adjust forests to a hotter and drier climate.

Box 2 **The framework of dryland mechanisms**

Dryland mechanisms are those processes affecting organism and ecosystem functions that have primarily developed in the dry/arid regions of the world (Grünzweig *et al*., 2022). They comprise adaptation and acclimation strategies of organisms, collectively forming a complex syndrome of ecosystem traits for coping with permanent or seasonal dryness at both the organismal and ecosystem levels. They are primarily driven by dryness, heat, nonrainfall water sources (such as dew and fog), water pulses, and/or intense solar radiation. Climate‐smart forestry (CSF) might simulate such naturally occurring mechanisms proactively as an adaptive management measure for temperate forests. An example of a relevant mechanism that could be promoted by CSF in regions subjected to increasingly frequent extreme droughts is hydraulic redistribution, a process by which deep‐rooted trees passively transport water through their root systems from deeper, moister soil layers to shallower, drier ones. In addition, shaping the forest surface structure might optimise the ability of air cooling through convective heat flux. CSF could create the boundary conditions for these mechanisms to become effective (for a detailed description of each mechanism, see Grünzweig *et al*., 2022). In Section IV, we provide detailed examples of dryland mechanisms and measures for their potential use.

**Fig. 2:**
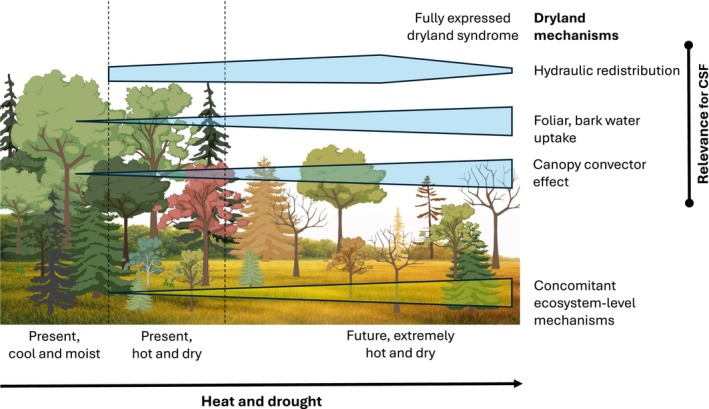
Conceptual model of the expected role of dryland mechanisms along a climatic gradient from cool and moist towards hot and dry conditions (*x*‐axis), and their relevance for climate‐smart forestry (CSF, *y*‐axis). Hypothetical changes in the state and structure of a temperate forest along the gradient are depicted in the background. Under present moist conditions, many dryland mechanisms are of minimal importance. Still, their importance rises considerably during current climate extremes, and they are expected to reach a maximum towards an extreme future climate (represented by the height of the bars along the heat and drought gradient). The blue bars show the expected activity of the three main dryland mechanisms suggested as potential targets for CSF (see Section IV). The yellow triangle indicates a series of dryland mechanisms that are expected to operate simultaneously with the CSF‐targeted mechanisms (see text). Background illustration created with BioRender.com (https://BioRender.com/25lih5j), based on an image by ChatGPT.

For our deliberations, we position the dryland mechanisms described by Grünzweig *et al*. (2022) within a gradient of intensifying hot and dry conditions, and assess their potential exploitation in CSF to proactively enhance forest resistance and resilience to drought (Fig. 2). The main dryland mechanisms we suggest as targets for CSF are hydraulic redistribution, foliar and bark water uptake, and the canopy convector effect, which enhances convective heat loss. These mechanisms can be targeted by forest management through tree species selection and stand structural measures (see Section IV), and are expected to already take effect under less extreme conditions (Fig. 2).

Some mechanisms will act only under extreme drought conditions and operate at the ecosystem level during climate extremes (cf ‘concomitant ecosystem‐level mechanisms’ in Fig. 2) and include soil hydrophobicity (water repellence), horizontal redistribution of resources (e.g. water, soil, litter, and seeds), and abiotic degradation of dead organic matter (photochemical and thermal degradation), all of which modify carbon and nutrient cycling, surface properties, and soil water retention. They are not directly controllable by management, and thus, we do not further consider them in Section IV.

Management practices that include dryland mechanisms need to balance – as always – benefits under extreme conditions, which will still be rare for many forests at present, and not necessarily the prevailing conditions in the future, with potential disadvantages during favourable periods.

In the following sections, we first summarise current knowledge on the impacts of heat and drought on tree carbon, nutrient, and water relations and how changes in tree physiology affect their growth, functioning, and survival. We also consider how the temporal course and intensity of such extreme events affect physiological traits.

We then explore how the alteration of different forest characteristics by management interventions that are frequently adopted to mitigate the effects of drought may affect these key ecophysiological processes and, thus, growth and mortality trajectories. Additionally, we discuss how CSF in temperate regions can learn from arid forests by actively promoting dryland mechanisms.

By doing so, we provide a mechanism‐based critical assessment of forest management practices with the goal of guiding and supporting future CSF in response to changing environmental and climatic conditions (following the suggestion of Cooper & MacFarlane, 2023). We do not offer simple fixes, but instead present a thorough discussion of possible strategies and the multiple associated factors that would need to be considered in actual management decisions. Our work focuses on forest management practices typical of temperate, Mediterranean, and boreal regions; however, the mechanism‐based recommendations should be applicable to any other forest type.

## II. Climate change and its impact on the ecophysiology of trees and forests

### 1. Drought and heat

Drought is expected to increase in frequency and intensity in the future, with substantial impacts on the hydraulic system, photosynthesis, and nutrition of trees (Mcdowell *et al*., 2008; Gessler *et al*., 2017). Geographical variations in climate change must be considered, as impacts vary substantially with altitude, latitude, and distance from the ocean; mountain regions, higher latitudes, and continental interiors experience more pronounced changes (IPCC, 2021). These spatial variations directly influence the severity of drought and heat stress.

Low soil water availability and atmospheric drought (i.e. increased water vapour pressure deficit (VPD)) decrease tree water potential and lead to a cessation of tissue growth (Zweifel *et al*., 2021) because plant cells must maintain turgor (positive water potential) for cell activity and particularly cell expansion (Steppe *et al*., 2015). Cell growth is generally more sensitive to water stress than photosynthesis (Hsiao, 1973), and evidence from experiments and modelling across a range of herbaceous and woody plants consistently demonstrates that growth ceases before photosynthesis, leading to the accumulation of nonstructural carbohydrates (NSCs; McDowell *et al*., 2011a, Muller *et al*., 2011). Exceptionally prolonged droughts can reduce starch stores across the major tree organs, while short but intense droughts are unlikely to induce much carbon shortage before the trees experiencing hydraulic failure. Drought impacts increase with water stress because of increased demand for sugars for osmotic regulation and slowed phloem transport throughout the plant (McDowell *et al*., 2011a). Periods of high NSC concentrations may cue greater investment into defence or increased transfer to the mycorrhizosphere microbiome, facilitating acclimation to the adverse conditions (Prescott *et al*., 2020).

Prolonged and severe droughts can directly lead to hydraulic failure (McDowell *et al*., 2011a). Loss of water supply to the tree, ultimately leading to lethal desiccation, may occur due to xylem embolism (Milburn, 1966; Milburn & Johnson, 1966; for a detailed description of the mechanisms of embolism formation see Tyree & Sperry, 1989 and Tyree & Zimmermann, 2002) or loss of hydraulic connection at the soil–root interface (Carminati & Javaux, 2020). Tree hydraulic systems can acclimate to drought by forming smaller xylem vessels, which are less vulnerable to cavitation, but at the expense of lower water transport efficiency (e.g. Fonti *et al*., 2013; Guet *et al*., 2015).

Concerning tree stature, large, tall, and dominant trees seem to be most negatively affected by drought (Bennett *et al*., 2015; Grote *et al*., 2016), alongside seedlings and saplings that do not yet have a well‐developed root system. In addition, any tree with a mal‐acclimated rooting system may be vulnerable to drought (see Section III.3). The drought sensitivity of tall trees is primarily attributed to their higher inherent hydraulic vulnerability and the exposure of their crowns to greater evaporative demand in the upper canopy. At sites with low precipitation, the height‐to‐diameter ratio of trees is typically lower than forests receiving high precipitation (Reis *et al*., 2018), suggesting that the balance between tree height and sapwood area may be crucial for drought resistance and a criterion for management activities (see Section III.4).

Water availability also affects whole‐tree carbon and biomass allocation and, thus, in turn, access to soil water resources. According to the sink control theory of the plant's carbon balance (Gessler & Zweifel, 2024), low growth rates resulting from atmospheric and soil drought limit the carbon sink strength of trees as fewer carbohydrates are needed for cell and cell wall production. Moreover, drought may impair the rhizosphere microbiome, leading to lower sink demand, and consequently reduced assimilate transport belowground, with potentially negative feedbacks on photosynthesis (Joseph *et al*., 2020). By contrast, carbon and biomass allocation to the belowground compartments may also increase during drought, following the functional partitioning theory that postulates that plants allocate biomass primarily to those organs that acquire the most limiting resource(s) (Poorter *et al*., 2012). Differences in drought intensity and duration (as described in the previous section) can explain these divergent observations: initially and during mild water limitation, when cell expansion is still functional, carbon allocation to roots may facilitate water uptake and overall root functioning (Schönbeck *et al*., 2020). When water limitation intensifies, root‐rhizosphere metabolic activity and growth cease and assimilate transport declines. Only recently, Shakas *et al*. (2025) have found that variable water availability in the past may co‐shape biomass allocation to roots. While permanently drought‐exposed trees were unable to increase carbon allocation to roots, they were able to develop a deep rooting system during 10 yr of irrigation, which allowed them to exploit deep water resources after the cessation of irrigation. Although this appears to conflict with the functional partitioning hypothesis, including ecological memory (*sensu* Walter *et al*., 2013; Hilker *et al*., 2016) as a mechanism for integrating a tree's long‐term experience makes this strategy effective for optimising resource acquisition. This example suggests that forest management considerations should take into account longer‐term environmental conditions and ecological memory.

Viewed from the source side of carbon relations (see Gessler & Zweifel, 2024), assimilate availability can decrease during drought, caused by stomatal closure. Maintenance metabolism and cellular repair depend on a continuous supply of carbohydrates, which can be temporarily provided by remobilising stored compounds. Carbon starvation, indicated by the reduced availability of NSCs, describes the situation in which the carbon demand for maintaining cellular and defensive metabolism is not met due to a low supply from photosynthesis and storage (Mcdowell *et al*., 2008), causing, together with hydraulic failure, drought‐induced mortality (Adams *et al*., 2017).

Fertilisation is known to reduce trees' resistance to drought (Linder *et al*., 1987). High soil nutrient availability (often occurring at sites with a normally sufficient soil water supply) favours, according to the functional partitioning theory, preferential biomass allocation aboveground at the expense of the rooting system and may thus predispose trees to growth reduction or even mortality during extreme drought events (Gessler *et al*., 2017). This may be a factor also to account for when managing for tree diversity (see Section III.2) and when applying thinning treatments (see Section III.3).

The defence potential of plants is not only affected by C but also by nutrient availability (Dutta *et al*., 2024), and thus, nitrogen (N) availability is an essential factor for the tree's defence potential (Gessler *et al*., 2017) during drought events. In addition, nutrient supply also affects other plant functions central for the adjustment to drought, such as water use efficiency (Querejeta *et al*., 2022). Higher NSC availability under mild drought (as described in the previous section) may also increase C transfer to rhizosphere microorganisms (Lv *et al*., 2023), thereby optimising nutrient uptake and compensating for reduced ion mobility at lower soil water content. Thus, drought intensity and duration may affect plant nutrient status and related functions. After a drought, sites with high nutrient availability may allow plants to restore their nutrient allocation more quickly, thereby promoting recovery (Gessler *et al*., 2017). These partially opposing impacts of nutrient availability need to be considered when applying CSF on sites with varying fertility and when implementing nutrient management (see Section III.5).

Heat, that is temperatures exceeding the optimum for photosynthesis (*T*_opt_), which is rarely above 30°C in C_3_ plants (Kumarathunge *et al*., 2019; but see Yamori *et al*., 2014), can reduce net carbon assimilation by inhibiting RuBisCO activase, along with an increase in dark and photo‐respiration (Rennenberg *et al*., 2006). However, thermal acclimation allows plants to adjust the photosynthesis optimum to higher temperatures (Kumarathunge *et al*., 2019) so that for many plant species, the ratio between photosynthesis and respiration is homeostatic across a wide range of temperatures (Dusenge *et al*., 2019). Heat may lead to lower carbon sequestration in forests mainly due to increased ecosystem respiration (*R*_eco_) and carbon loss, especially from the soil (Duffy *et al*., 2021). Even though temperature acclimation of autotrophic (Atkin & Tjoelker, 2003) and apparent thermal acclimation of heterotrophic respiration (He *et al*., 2023) can occur, at higher temperatures respiration rates continue to rise, in contrast to sharply declining rates of photosynthesis (Duffy *et al*., 2021).

The magnitude of these effects and thus the reduction in C sequestration depend on the type of forest ecosystem, the seasonal dynamics of growth and photosynthesis, and the timing of extreme events (Melillo Jerry *et al*., 2011; Nottingham *et al*., 2020; Xu *et al*., 2020).

Exceedance of the leaf critical temperature, which is species‐specific and ranges between *c*. 45 and 47°C (Esperon‐Rodriguez *et al*., 2021), can lead to leaf and canopy damage in the upper canopy. However, such ‘scorching’ will mainly occur when extreme heat and drought (the latter impairing transpirational cooling) co‐occur.

Extreme heat can also lead to increased cuticular transpiration as water loss shifts from stomatal to cuticular pathways, with this thermal stress creating persistent ‘leaky legacy’ effects that compromise the cuticular barrier and contribute to hydraulic failure even after temperatures return to normal (Marchin *et al*., 2022; Fernandes *et al*., 2025).

As hot droughts are becoming more frequent (Allen *et al*., 2015), air‐cooling by convective heat flux may become more important in the future (see Section IV).

#### Compound effects

Atmospheric evaporative demand increases nonlinearly with increasing air temperature (Seneviratne *et al*., 2010; Grossiord *et al*., 2020a). Hot droughts, characterised by high potential evapotranspiration (ET) and low soil water availability, are most detrimental for forests and can be an important driver of tree mortality globally (Allen *et al*., 2015). Even without widespread mortality, hot droughts can cause temperate ecosystems to shift from carbon sinks to sources, as seen during the hot and dry growing season of the year 2003 that significantly reduced gross primary productivity in European low‐elevation forests (Ciais *et al*., 2005). The hot drought in the summer of 2018, when low soil water availability and high VPD co‐occurred, affected large areas in Europe, causing widespread hydraulic failure and early leaf senescence also in individuals of tree species that had been thought to be heat‐ and drought‐resistant (Schuldt *et al*., 2020; Neycken *et al*., 2025). This points to the fact that temperate and boreal forests may have approached the point of substantial ecological and economic transition (cf Section III.1; Wessely *et al*., 2024). McDowell *et al*. (2018) showed that rising temperature and VPD, together with increasing frequencies and severities of droughts, which all are triggering carbon starvation and hydraulic failure, can explain increasing mortality rates in moist tropical forests, indicating the global impacts of climate change as well as a similarity of the mechanisms inducing damage. It is more and more acknowledged that increasing VPD and thus atmospheric drought is clearly related to growth reduction and increasing tree mortality, exacerbating the effects of reduced soil water availability (Breshears *et al*., 2013; Trotsiuk *et al*., 2021; Hunziker *et al*., 2022).

Heat and drought, as well as precipitation variability within and between years, increase fire risks in many Mediterranean, temperate, and boreal forests (e.g. Ruffault *et al*., 2018; Wasserman & Mueller, 2023). While periods of high precipitation can increase biomass and, hence, available fuel (Jin *et al*., 2014), air temperature is often correlated with fire extent and severity, and relative humidity controls fuel moisture and, consequently, fire behaviour (e.g. Brown *et al*., 2004; Westerling *et al*., 2011). Therefore, CSF should consider the increasing fire risk and develop strategies for its mitigation (see Section III.2).

Climate warming is also extending growing seasons and advancing spring phenology for many tree species, but this can create increased vulnerability to late spring frosts, which in turn reduce photosynthetic productivity and delay subsequent phenological stages, creating cascading effects on forest carbon cycling and productivity (Wang *et al*., 2025). Frost sensitivity thus needs to be considered when selecting tree species for CSF (see Section III.1).

### 2. Abiotic effects on the interaction between trees and pests and pathogens

Secondary metabolites enable trees to defend themselves, for example, against bark beetles and associated microbes (Huang *et al*., 2020). Experimental evidence shows that drought periods reduce resin‐based defence in trees, which is strongly based on secondary metabolites (Netherer *et al*., 2015), even though acclimation to long‐term reduced water supply seems possible (Rissanen *et al*., 2021). Here, the balance between drought impact on growth (sink demand) and photosynthesis (sink activity; Cabon, 2026), which depends *inter alia* on drought intensity (see Section II.1), seems crucial.

Thus, mild drought might increase the carbon resources available for defence. However, the composition of defence compounds may be affected, depending on changes in N vs C availability (Herms & Mattson, 1992). However, the high metabolic cost of multiple stresses (e.g. drought and pathogens) might still lead to a depletion of defence compounds and to insufficient allocation to sink tissues (Mundim & Pringle, 2018). However, under long and intensive drought, reduced availability of NSC (McDowell *et al*., 2011a), as well as of nutrients (Gessler *et al*., 2017), may limit C and N allocated to defence compounds (Thompson *et al*., 2024). In response to pathogen attacks, the development of reaction zones and various other pathogen‐induced defence mechanisms incurs significant carbon costs, thereby elevating the consumption of NSC. If intensive drought already reduced NSC availability, this will consequently accelerate drought‐induced mortality (Oliva *et al*., 2014). Conversely, considerable drought‐induced carbon depletion may render it impossible for the tree to invest sufficiently into defence, such that it may succumb to pest attacks.

Furthermore, vascular wilt pathogens (fungi, bacteria, and oomycetes) spreading through xylem vessels can block water transport (Yadeta & Thomma, 2013) and aggravate heat‐ and drought‐induced hydraulic impairment. During infection of xylem vessels with fungal pathogens, the resulting tylosis production in the xylem can reduce water transport even further and contribute to hydraulic failure (Bortolami *et al*., 2019). Although insect and pathogen infestations can occur without heat or drought events, mutually reinforcing interactions are known to take place (Seidl *et al*., 2017; Jactel *et al*., 2019; Trugman *et al*., 2021). On the one hand, pathogens may exacerbate the direct effects of heat and drought, leading to longer‐term legacies of mortality and growth depression. On the other hand, climatic extremes weaken trees, making them susceptible to biotic attacks.

Multivoltine insect species such as bark beetles (e.g. *Ips typographus*) can have more generations in a warmer climate than in the past (Singh *et al*., 2024), increasing the pressure on the host trees and extending the area of infestation towards higher latitudes and altitudes (Hartmann *et al*., 2025a). However, as found for gypsy moth infestation, climatic drivers can strongly interact with forest structure and species composition (Hentschel *et al*., 2018), and thus, the management may provide effective mitigation strategies (see Section III.2). Fungal pathogens can also benefit from higher summer temperatures, especially under humid conditions, thereby increasing infection pressure under global warming, particularly for trees weakened by preceding drought events (Hietala *et al*., 2018; Brodde *et al*., 2019).

## III. Climate‐smart management practices and their ecophysiology‐based impacts on forest resistance and resilience to climate change

Climate change can affect the functioning of trees and forest ecosystems in different ways. Note that we define ecosystems according to Eugene Odum as ‘any unit that includes all organisms in a given area interacting with the physical environment such that the flow of energy leads to clearly defined trophic structure, biotic diversity, and material cycles’. Forests and forest ecosystems are used as synonyms here and are ecosystems dominated by trees (according to the FAO Forest Resource Assessment, Working Paper 194). The spatial scale of ecosystems we are focusing on is the forest stand level.

Adverse effects of climate change are conveyed directly through a restricted water, carbon, and nutrient balance, and indirectly by improving the environmental conditions for pests and pathogens, while simultaneously impairing the trees' defence mechanisms. While climate change may have positive effects on some forest functions in particular regions (cf Spathelf *et al*., 2014; Luo *et al*., 2020), it more often jeopardises the provision of ecosystem services. Thus, CSF should specifically target mitigating direct and indirect adverse effects and increasing potential climate change‐related benefits. Moreover, CSF could manage forests in a way that dryland mechanisms (Box 2) are promoted proactively for whole‐ecosystem drought acclimation.

**Fig. 3:**
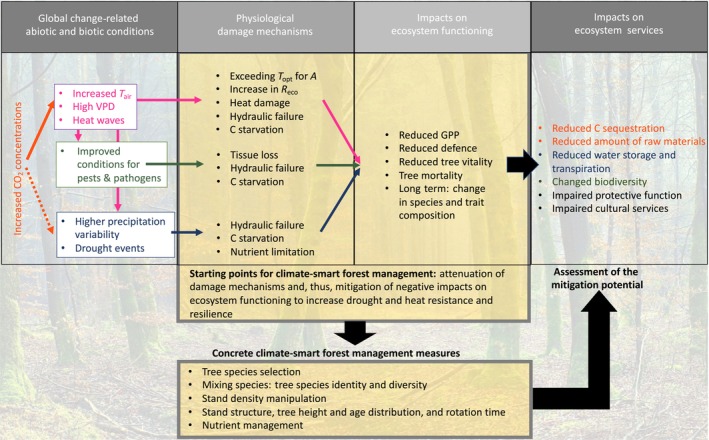
Ecophysiology‐based concept for climate‐smart forestry (CSF). Changes in biotic and abiotic conditions due to climate change (temperature‐related (magenta), water availability‐related (dark blue), and pests and pathogens (dark green) affect the physiology of trees, conveying different damage mechanisms. Such physiological damages concertedly impact ecosystem processes and functioning, with repercussions for the services that forest ecosystems provide to humanity. Therefore, the main starting point for CSF should be mitigating the physiological damage mechanisms. For such an approach, it is imperative to comprehend how specific management options influence individual‐tree and integrated stand ecophysiology, as well as their mitigation potential regarding losses in ecosystem services. The colours for ecosystem services indicate negative feedback on atmospheric CO_2_ concentration (orange), the water cycle (dark blue), and pathogen damage (dark green). *T*_opt_ for A, the optimal temperature for photosynthesis; *R*_eco_, ecosystem respiration; GPP, gross primary production. High *T*_air_ can also lead to high water vapour pressure deficit (VPD) of the air, resulting in atmospheric drought. We acknowledge that some factors, such as increased *T*_air_, might also positively affect tree and forest functioning in areas and ecosystems that are temperature‐limited (mainly in boreal and alpine regions). However, hot‐drought‐induced tree mortality has also been observed in the boreal areas (Hammond *et al*., 2022).

The primary management target should be to attenuate the physiological damage mechanisms related to heat and drought and their concerted impacts on ecosystem functioning (Fig. 3). If by these means the resistance and resilience of forests can be increased, ecosystem services, including carbon sequestration, which contributes to climate change mitigation, may be maintained and even increased.

We propose that concrete climate‐smart forest management measures should be developed based on the primary target mentioned above. This review will explore suitable tree species selection (Section III.1), tree species mixture (Section III.2), stand density manipulation (Section III.3), stand structural management measures (Section III.4), and nutrient management (Section III.5) for their potential to increase resistance and resilience of forests. We must emphasise that current and future CSF applications (and also field trials with controlled designs) need to continuously assess the mitigation potential to allow for readjustment and optimisation of management in response to the constantly changing climate.

We acknowledge that climate change may also increase wind‐ and fire‐driven disturbances. However, the physiological damage mechanisms shown in Fig. 3 are only indirectly related to these disturbances. For fire, heat‐ and drought‐induced tree mortality may change fuel load and thus the fire regimes, leading to more intense and potentially larger fires (Stephens *et al*., 2018). Moreover, heat and drought can change resin and terpene production (Holopainen *et al*., 2018), potentially affecting vegetation flammability (Pausas *et al*., 2016). Drought conditions have been demonstrated to compromise the structural integrity of trees, thereby increasing their susceptibility to stem breakage when subjected to wind loads (Csilléry *et al*., 2017). Owing to these indirect relationships, neither of these two disturbance types is central to our review; however, they are mentioned when CSF practices influence the resistance or resilience of forests to wind or fire.

### 1. Tree species selection

Due to the velocity of changes in climate, empirical knowledge of forest practitioners will often lose accuracy (von Detten & Faber, 2013) or, as phrased by the Scientific Advisory Council for Forest Policy at the German Federal Ministry of Food and Agriculture (Bauhus *et al*., 2021), ‘rapidly advancing climate change is accelerating the erosion of the relevance of previous empirical knowledge and leading to an increase in uncertainties’. Therefore, empirical knowledge must be supplemented with scientific evidence and scenario‐based projections to enable forest managers to make informed decisions regarding tree species selection and assisted migration options (Zischg *et al*., 2021; Chakraborty *et al*., 2024b). Assisted migration comprised the following: (1) assisted population migration, also known as assisted genetic migration or gene flow: moving populations (= seed sources/provenances) to new locations within the historical species range; (2) assisted range expansion: moving species or populations from their current to suitable future ranges just beyond the historical species range, facilitating or mimicking natural dispersal that would otherwise occur over centuries; and (3) assisted species migration: moving species or populations to a location far outside the historical species range, where they could not arrive through natural processes or only over millennia (definitions adopted from Chakraborty *et al*., 2024b).

#### Ways to attenuate the physiological damage mechanisms (in order to strengthen tree and ecosystem resistance and resilience)

Some tree species and provenances (genetically adapted to local environmental conditions) are better suited to future climatic conditions at a given site than others (Niinemets & Valladares, 2006; Leites & Benito, 2023). Some tree species (e.g. Vilagrosa *et al*., 2012) and provenances (e.g. Stojnić *et al*., 2017) from dry regions show, for example, higher embolism resistance. This evolutionary adaptation is related to functional and structural traits that can be selected for assisted migration.

Table 1 shows traits that are indicative of resistance against embolism: wood density is often negatively correlated with vessel size; smaller vessels increase resistance to cavitation and are thus less prone to hydraulic failure and loss of hydraulic conductance (Chave *et al*., 2009). Trees with short xylem vessels of small diameter, characterised by small and few pits, hence exhibit more negative *P*_50_ values (Xylem pressure at 50% loss of hydraulic conductance), enabling them to maintain water transport under lower water potentials. For selecting a suitable tree species for a given site, the difference between the minimum water potential a tree experiences there and its *P*_50_, that is the hydraulic safety margin, could be considered for mitigating the risk of hydraulic failure.

**Table 1:** Key morphological and physiological traits that influence tree responses to drought stress.

Trait	Adaptive significance for drought conditions	References
Specific leaf area (SLA)	Lower SLA indicates thicker leaves with more structural tissue and higher leaf mass per area, reducing water loss through increased boundary layer resistance and often correlating with longer leaf lifespan and greater drought tolerance	Wright *et al*. (2004); Poorter *et al*. (2009)
Stomatal density and size	Lower stomatal density can reduce maximum transpiration rates under dry conditions, while smaller stomata allow faster responses to changing conditions, improving water use efficiency	Franks & Beerling (2009)
CO_2_ stomatal sensitivity	Species with higher stomatal sensitivity to elevated CO_2_ show greater reductions in stomatal conductance, leading to improved water use efficiency and reduced transpiration under future atmospheric conditions, providing advantage during drought periods	Medlyn *et al*. (2001)
Xylem pressure at 50% hydraulic loss (*P*_50_)	More negative *P*_50_ values indicate greater resistance to drought‐induced embolism and hydraulic failure, allowing trees to maintain water transport under lower water potentials	Anderegg *et al*. (2016a)
Sapwood‐to‐leaf area ratio (*A*_s_ : *A*_l_)	Higher *A*_s_ : *A*_l_ ratios provide greater hydraulic capacity to supply water to leaves, buffering against drought stress and reducing risk of hydraulic failure	Mencuccini & Grace (1995)
Root‐to‐leaf area ratio (*A*_r_ : *A*_l_)	Higher *A*_s_ : *A*_l_ ratios improve water uptake capacity relative to transpirational demand, enhancing drought avoidance through maintained water supply	Mokany *et al*. (2006)
Wood density	Higher wood density often correlates with narrower vessels, greater mechanical strength, and increased resistance to drought‐induced cavitation	Chave *et al*. (2009)
Turgor loss point (TLP)	More negative TLP values allow leaves to maintain turgor and physiological function at lower water potentials, extending the operational range under drought	Bartlett *et al*. (2012)
Hydraulic safety margin (HSM)	Larger HSM (difference between minimum water potential a plant experiences and *P*_50_) provides buffer against hydraulic failure during drought events	Choat *et al*. (2012)
Leaf size	Smaller leaves have thinner boundary layers, improving convective cooling and reducing leaf temperatures under hot, dry conditions	Wright *et al*. (2017)
Rooting depth	Drought‐sensitive species root more shallowly than drought‐resistant species	Feng *et al*. (2023)

It is essential to consider that there is often a trade‐off between the adaptation potential to more extreme conditions and the general efficiency of processes under milder ones. Wood density, for example, is frequently associated with narrower vessels and thus a higher cavitation risk; however, it also reduces water transport efficiency under high water availability. Another example is that higher *A*_r_ : *A*_l_ ratios improve water uptake in relation to transpiration loss, but require a greater investment of carbon in the root system.

However, there is often a trade‐off between cavitation resistance of the hydraulic system and water transport efficiency (Markesteijn *et al*., 2011; Grossiord *et al*., 2020b), which may lead to lower maximum photosynthesis at a given xylem cross‐sectional area (or more precisely, sapwood area) and thus stem dimension. The resistance and resilience of the stand to periods of drought may, therefore, come at the cost of reduced carbon uptake and growth rates during periods of high water availability. Given that drought‐ and heat‐induced tree mortality also strongly reduces forest stand C sequestration, and even turns an ecosystem from a C sink to a source, and affects the forests' and landscapes' water balance for extended periods (Anderegg *et al*., 2016b), these costs may appear acceptable. Still, they must be accounted for when establishing forest stands with more drought‐resistant species, balancing this with the economic and ecological risks of business‐as‐usual forest management.

Plant species also differ in stomatal sensitivity to elevated CO_2_, affecting water use efficiency and drought adaptation potential under future atmospheric conditions (Medlyn *et al*., 2001; Table 1), which may be a selection criterion for assisted migration and tree species selection in general.

Trees originating from warmer climates are often assumed to be adapted to higher temperatures and thus to exhibit a higher optimum temperature (*T*_opt_) for photosynthesis, implying that they would continue sequestering carbon at higher air temperatures. However, recent research has shown that phenotypic plasticity and acclimation play a larger role than long‐term evolutionary processes in adjusting *T*_opt_ (Sendall *et al*., 2015; Kumarathunge *et al*., 2019). Consequently, the temperature at the origin of a species or provenance is not necessarily indicative of its photosynthetic functioning under future climatic conditions. In addition, carbon uptake and growth are affected not only by photosynthetic capacity but also by direct environmental effects on sink activity (Gessler & Zweifel, 2024). Consequently, not only the T_opt_ of photosynthesis but also temperature requirements (Piovesan *et al*., 2023) and water supply for heterotrophic metabolism, such as cell division and expansion, must be met. To our knowledge, there is no extensive research on the importance of adaptation vs acclimation for the temperature optima of cambial activities, which could inform tree species selection for CSF. However, some European tree species have been classified according to their growth sensitivity to prevailing hydrological conditions (Zweifel *et al*., 2021). The authors observed that VPD, and hence atmospheric drought, is the most critical limitation on growth during the diurnal course. The species‐specific VPD thresholds for growth of the five species analysed (*Abies alba, Fagus sylvatica, Fraxinus excelsior, Picea abies, Pinus sylvestris, Quercus petraea, and Quercus pubescens*) ranged within a narrow band of values for all species, supporting growth only at night. However, the soil water potential (SWP) threshold which allows growth given that the VPD conditions are favourable, varies considerably across species. *Quercus pubescens* grew at the lowest SWP, and *P. sylvestris* was the second most drought‐tolerant species, whereas *A. alba* required moist soil conditions for growth. Consequently, known sensitivities to hydraulic constraints and growth in response to soil and atmospheric drought should be criteria for site‐specific tree species selection.

#### Implications for climate‐smart forestry

Any tree species planted today must be capable of withstanding current and future climatic conditions. During the assisted migration of species from drier, warmer origins to temperate and boreal ecosystems, their frost sensitivity is one of the crucial environmental factors to be considered (Vitasse & Rebetez, 2018; Zohner *et al*., 2020). Recent estimates based on species distribution models (SDM) for different European regions that accounted for the continuous climate suitability during a tree's lifespan (i.e. the suitability today and under future climate conditions), consequently project a climate change‐induced tree species bottleneck for forest management (Wessely *et al*., 2024).

**Fig. 4:**
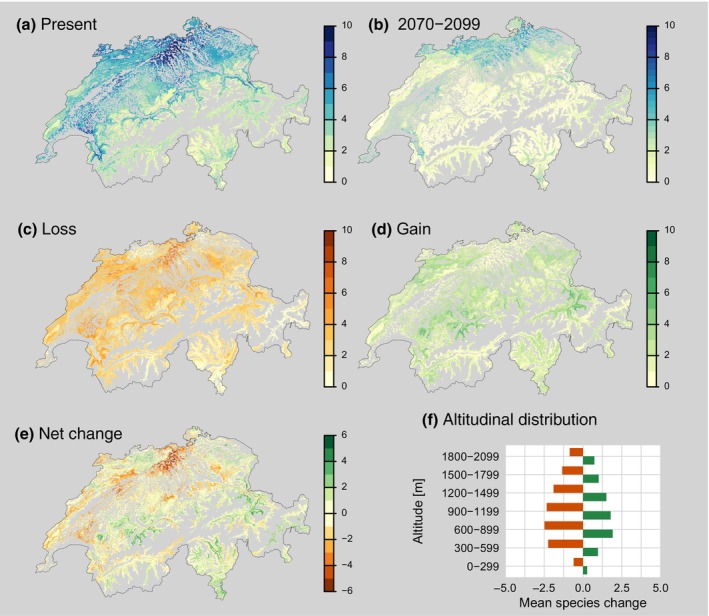
Present potential distribution of the 10 most abundant native forest tree species in Switzerland and changes to be expected in the future as calculated by an ensemble of species distribution models. (a) The number of species that have potentially suitable conditions at a given pixel under present climatic conditions. (b) The number of species suitable under today's climate and by the end of the century, assuming the RCP 8.5 scenario. (c–e) The gross loss, gross gain, and net change in the number of species, respectively. (f) The altitudinal distribution of losses and gains in the number of species. The colour codes indicate the number of species. The methodology for modelling the potential ranges is explained by Gessler *et al*. (2024), and the species list is given in Supporting Information Table S1. [Correction added on 9 March 2026, after first online publication: panels (b–d) have been updated to correspond with the figure legend and the main text.]

While aiming to project the fundamental niche of a species into future conditions, SDMs do not explicitly consider the physiological mechanisms responsible for climatic habitat suitability or species demography (establishment and mortality). Still, implicitly, these mechanisms and the related traits form the basis of a species' persistence at a given site (e.g. Kearney & Porter, 2009). Although the literature consistently calls for integrating physiological information more explicitly into SDMs to improve their predictive accuracy (Evans *et al*., 2015), the model outputs may be seen as integrating and balancing various aspects of site‐specific evolutionary adaptations. Fig. 4 shows the difference between the presence of the 10 most abundant and economically important tree species in Switzerland today and their availability for forest management in a dynamically changing climate in the future. We used an SDM ensemble (see Gessler *et al*., 2024) to determine the potential distribution of the 10 most common Swiss native forest tree species under current climate conditions. In addition, we calculated for the end of the century (2070–2099) the pool from these species that is suitable under both the present and future conditions under RCP 8.5 (business‐as‐usual scenario). For each location on the map, we then calculated the loss, gain, and net change in species. Our exercise, using Switzerland as an example, shows a general reduction in the climate suitability of the most abundant native tree species that can be used (and are currently favoured by forest managers) for establishing new forests. Main gross losses and negative net changes were observed in the Swiss plateau. In this region, the pool of common native species that find suitable climatic conditions may become locally very small, necessitating consideration of presently rare native species that are not currently focused on by forest management. Fig. S1 illustrates the future availability, as well as the losses and gains, for 38 tree species native to Switzerland. The figure shows that when accounting also for rarer tree species, the changes in the pool size of tree species available for silviculture are less alarming. Furthermore, it is essential to consider that many tree species will show a lagged response to climate change, such that they may still be present in a landscape (cf Huber *et al*., 2021; Fastovich *et al*., 2025) even though an SDM may feature them as not climatically suitable; hence, the SDM picture is likely to be on the pessimistic end of estimations regarding the set of prevailing tree species.

Net gains are indicated mainly in some inner‐alpine regions, while the most considerable losses are to be expected below 1200 m when we consider the 10 most abundant species (Fig. 4f). However, when accounting for the complete native species pool, a net tree species gain is projected between 600 and 1800 m.

In addition to rare native tree species, non‐native ones may also be considered in future management plans. Species not native to a given area, although potentially suited under a future climate, may negatively impact species interactions and biodiversity beyond trees in forests, or even become invasive (Wohlgemuth *et al*., 2022). Both effects can have a negative impact on the provision of ecosystem services. Moreover, country‐specific legal regulations (Pötzelsberger *et al*., 2020) restricting the introduction of non‐native tree species may apply. However, in areas that will face a substantial native tree species bottleneck for forest management (Wessely *et al*., 2024), non‐native species could be part of suitable CSF options. Generally, a careful consideration of the advantages and disadvantages of introducing non‐native species is necessary, also taking into account the impact on nature conservation.

Projections done with SDMs, as shown in Fig. 4, describe an assumed future niche equilibrium (Peñalver‐Alcázar *et al*., 2021) under the then‐given climatic conditions. This is not the same as the state of ecosystems in the respective period of the climate change scenario, as ecosystems and their constituent tree species exhibit highly dynamic behaviour (Hsu *et al*., 2025): competitive interactions between species shift with changing environmental conditions, successional dynamics play an important role, where early colonisers may facilitate or inhibit later species establishment. In addition, disturbance regimes (fire, wind, and pests) can create opportunities for species turnover that are independent of climate suitability, and multi‐cohort dynamics in mixed‐species forests can lead to complex temporal dynamics. These limitations become particularly evident when SDMs are applied at the stand level, where local‐scale dynamics may override broad climate suitability patterns (Anselmetto *et al*., 2025).

Web‐based apps that are starting to become available for the selection of tree species targeted to forest practitioners. Considering (different) climate projections, the SDM‐based habitat suitability of species, and the local management context (e.g. https://tree‐app.ch for Switzerland; https://www.klimafitterwald.at for Austria; https://www.fs.usda.gov/nrs/atlas/tree/ Climate Change Tree Atlas for East USA; MacKenzie & Mahony (2021) for British Columbia; Boisvert‐Marsh *et al*. (2022) for eastern Canada) may provide some guidance if the limitations mentioned above are accounted for.

An additional problem in predicting the suitability of tree species for a particular habitat is that climate extremes are usually not explicitly accounted for in SDMs, even though they may be more critical than average climatic conditions when projecting species range shifts (Germain & Lutz, 2020). Given these restrictions, we can only support the call by Evans *et al*. (2015) that ‘modellers, physiologists and […] practitioners [should] work collaboratively to build [new dynamic and mechanism‐based] models, interpret results and consider […] management options […]’. Until then, SDMs still provide some broad and general information on areas where particular tree species might become less (or more) suitable. By integrating the practical knowledge of forest practitioners with improved communication between science and practice on the potentials and limitations, SDMs and the Apps based on them can effectively assist in management decisions.

##### Key points for climate‐smart forest management

In addition to empirical knowledge of species selection, novel tools based on SDMs can provide a broader and scientifically supported basis for selecting the most suitable tree species for local conditions under a rapidly changing climate, when the inherent restrictions of the models are considered. Trade‐offs between resistance to drought and performance under normal conditions are often associated with specific trait combinations and should be accounted for. Where bottlenecks (*sensu* Wessely *et al*., 2024) for the most important native tree species for management and providing ecosystem services are to be expected (see Fig. 4 with Switzerland as an example), native rare species should be considered. In addition, non‐native species might be considered with due care.

### 2. Mixing species: tree species identity and diversity

Biodiversity is often assumed to generally improve ecosystem functioning and enhance the services ecosystems provide to humanity (Mace *et al*., 2012). The most important biodiversity effects driving ecosystem functioning include niche complementarity (where species differ in resource use strategies), facilitation (positive interactions that benefit species), selection effects (where particular species disproportionately influence ecosystem processes), and mass ratio effects (where dominant species' functional traits drive ecosystem properties; Turnbull *et al*., 2013; García‐Palacios *et al*., 2017). In forests, it has indeed been shown that the diversity of tree species stabilises productivity, increases drought tolerance, and diversity is therefore assumed to improve the ecosystem's adjustment potential to climate change (Schnabel *et al*., 2021).

Tree species richness not only changes how individual species may respond to drought and other stress factors. One of its central functions in forest management is the portfolio effect, namely to spread risks, including unknown ones, among species that respond differently to particular stress and disturbance factors so that only one or a few but not all tree species are affected (Bauhus *et al*., 2017). This reduces the probability of stand‐replacing disturbances and thus avoids the challenging situation of regenerating forest in large areas under extreme climatic conditions.

#### Ways to attenuate the physiological damage mechanisms

It has been demonstrated that increased tree species richness diminishes drought stress in plantation settings, an effect that was not only related to the desiccation tolerance of the individual species contributing to the mixtures (Jing *et al*., 2024). Niche complementarity and facilitation were identified as the two key mechanisms that enhance drought tolerance in diverse systems, even though an individual species may not be particularly adapted (Haberstroh & Werner, 2022). However, in addition selection effects may promote the presence and dominance of drought‐resistant species as tree diversity increases (Grossiord *et al*., 2020a,b). This example can provide complementary information to the selection of tree species (Section III.1). On the one hand, the mixture of species may be able to increase the drought resistance and resilience not only of the entire forest but also of the individual species (compared with their monocultures or natural plant communities; see Hajek *et al*., 2022). Consequently, the local habitat suitability for species with given sets of traits in ecosystems characterised by high planted tree diversity may be significantly greater than that predicted by SDMs, indicating a potential of CSF for mitigating climate change impacts. On the other hand, at the stand level, the higher probability of including drought‐tolerant species in high‐diversity forests may reduce the risk of loss of ecosystem services during drought periods (Bauhus *et al*., 2017).

Reality, however, is likely more complex: a global study indicated that higher species diversity enhanced drought resistance in only half of the global forests and highlighted high spatial variability (Liu *et al*., 2022). In general, it appears that the relationship between increased drought resistance or resilience and tree species diversity is not consistent across forest types (Grossiord *et al*., 2014), depends strongly on the environmental context (Ratcliffe *et al*., 2017), and is affected by the trait complementarity of the species in the mixture (Hajek *et al*., 2022).

In Table 2, we compiled findings from the literature (Web of Science, Core Collection) on whether tree species diversity mitigates the effects of heat or drought. Ten of the 27 publications demonstrated a positive impact of diversity on drought resistance, resilience, or recovery, whereas five primarily reported adverse impacts. In almost half of the papers, there were either no clear or mixed (positive and negative) effects. Accordingly, Forrester *et al*. (2016) concluded that drought stress can be, but is not generally, lower in mixed‐species forests; it may even vary between sites for a given species combination and may also change with stand age.

**Table 2:** Effects of tree species diversity on heat and drought resistance, resilience, and recovery.

Type of stressor	Species	Diversity gradients	Climatic region and climatic conditions	Resistance, resilience, recovery	Effect	References
Drought	*Acer pseudoplatanus, Aesculus hippocastanum, Fraxinus excelsior, Prunus avium, Sorbus aucuparia, Betula pendula, Carpinus betulus, Fagus sylvatica, Quercus petraea,* and *Tilia plathyphyllos*	1, 2, and 4	Temperate continental *P*: 484 mm *T*: 8.8°C	Resistance and resilience	No general effect on growth resistance and resilience Negative effect on ectomycorrhizal resistance and resilience, positive effect on resistance and resilience of arbuscular mycorrhiza	Sachsenmaier *et al*. (2024)
Drought	*Fagus sylvatica, Fraxinus excelsior, Quercus petraea, Tilia cordata, Picea abies,* and *Pinus sylvestris*	1, 2, 3, 5, and 6	Temperate *P*: 578 mm *T*: 9.4°C	Resistance	Positive biodiversity – productivity relationships during nondrought years turn negative during drought	Shovon *et al*. (2024)
Drought	Global dataset	1–58	Global dataset	Resistance	Higher species diversity could enhance drought resistance in about half of global forests but spatial variability is high	Liu *et al*. (2022)
Drought	*Populus tremuloides* and *Picea glauca*	1 and 2	Boreal *P*: 381 mm *T*: 2.3°C	Resistance and resilience	Mixtures of trembling aspen and white spruce were more productive under and resilient to drought than pure stands.	Cardoso *et al*. (2025)
Drought	*Fagus sylvatica, Quercus robur, Acer pseudoplatanus,* and *Carpinus betulus*	1, 2, and 3	Temperate *P*: 829 mm *T*: 10.7°C	Resistance	Growth resistance to drought of European beech was higher in mixtures than in monocultures	Vannoppen *et al*. (2020)
Drought	*Abies alba, Picea abies, Pinus nigra, Pinus sylvestris, Acer pseudoplatanus, Betula pendula, Carpinus betulus, Castanea sativa, Fagus sylvatica, Fraxinus excelsior, Ostrya carpinifolia, Quercus robur, Quercus petraea, Quercus cerris, Quercus faginea,* and *Quercus ilex*	1 up to 5	Gradient across Europe Hemiboreal‐Mediterranean	Resistance	The temperate beech and the thermophyllous deciduous forests showed an increase in drought resistance with increasing diversity. Such an effect was not observed in the hemiboreal, the mountain beech, and the Mediterranean forest.	Grossiord *et al*. (2014); Fig. 5
Drought	*Pinus sylvestris, Picea abies,* and *Betula pendula*	1, 2, and 3	Boreal *P*: 700 mm *T*: 2.1°C	Resistance	Negative relationship between drought resistance and resilience	Grossiord *et al*. (2013); Fig. 5
Drought	350 000 trees from 34 sites located in boreal, temperate, and Mediterranean and tropical biomes	1 and 2 or more	34 sites	Resistance (mortality)	Tree diversity increased survival of saplings of such species that showed high mortality in monoculture under drought	Blondeel *et al*. (2024)
Drought	*Abies alba, Fagus sylvatica,* and *Picea abies*	1–3	3 temperate sites *P*: 1399–1775 mm *T*: 5.1–7.2°C	Resistance, recovery and resilience	No effect	Gillerot *et al*. (2021)
Drought	*Acer platanoides, Acer saccharum, Betula papyrifera, Betula pendula, Larix decidua, Larix laricina, Picea abies, Picea pungens, Pinus strobus, Pinus sylvestris, Quercus robur,* and *Quercus rubra*	1, 2, 4, 6, and 12	Temperate *P*: 881 mm *T*: 11.6°C	Resistance	Negative species richness effect on Normalized Difference Vegetation Index (NDVI) but not on Plant Area Index (PAI)	Hajek *et al*. (2024)
Drought	*Fagus sylvativa, Abies alba,* and *Quercus pubescens*	1 and 2	Temperate (latitudinal and elevation gradients in the Alps) *P*: 790–2079 mm *T*: 5.4–10.7°C	Resistance and recovery	No diversity effect on growth resistance and recovery	Jourdan *et al*. (2019)
Drought	*Acer pseudoplatanus, Acer platanoides, Acer saccharum, Aesculus hippocastanum, Betula papyrifera, Betula pendula, Carpinus betulus, Fagus sylvatica, Fraxinus excelsior, Larix decidua, Larix laricina, Larix eurolepis, Larix kaempferi, Picea abies, Picea pungens var. glauca, Pinus pinaster, Pinus sylvestris, Pinus strobus, Prunus avium, Pseudotsuga menziesii, Quercus petraea, Quercus robur, Quercus rubra, Salix Loden, Salix Tora, Sorbus aucuparia, Tilia cordata,* and *Tilia platyphyllos*	1, 2, 3, 4, and 6	Temperate; seven sites across Europe *P*: 484–1336 mm *T*: 8.3–13.8°C	Resistance	Species diversity reduces drought stress (determined via intrinsic water use efficiency), independent of drought intensity and desiccation tolerance of species	Jing *et al*. (2024)
Drought	*Fagus sylvatica, Quercus ilex,* and *Picea abies* as target species; additional species in mixtures	Gradients	Temperate to Mediterranean 253 plots distributed on a 16 × 16 km grid across Italy	Recovery	Crown defoliation as an indicator of tree health increases with tree diversity. After drought diversity was beneficial for defoliation trajectories of *P. abies* and *Q. ilex*.	Iacopetti *et al*. (2019)
Drought	*Fagus sylvatica, Sorbus aucuparia, Carpinus betulus, Betula pendula, Quercus* spp., *Acer pseudoplatanus, Corylus avellana, Frangula alnus, Sambucus nigra,* and *Crataegus* spp. (10 most abundant)	Natural gradients	Temperate; 218 plots in Bavaria *P*: 540–1344 mm *T*: 4.8–9.4°C	Resistance	High sapling species diversity reduced sapling vitality under drought stress	Beloiu *et al*. (2020)
Drought	*Fagus sylvatica, Quercus robur,* and *Quercus rubra*	1, 2, and 3	Temperate *P*: 826 mm *T*: 10.5°C	Resistance, recovery, and resilience	No effect of tree diversity	Vanhellemont *et al*. (2019)
Drought	*Lithocarpus glaber, Schima superba, Daphniphyllum oldhamii, Quercus fabri, Cyclobalanopsis glauca, Castanea henryi, Castanopsis sclerophylla, Cyclobalanopsis myrsinifolia, Triadica sebifera, Liquidambar formosana, Castanopsis carlesii, Acer davidii, Sapindus saponaria, Nyssa sinensis, Quercus acutissima, Castanopsis fargesii, Choerospondias axillaris, Cinnamomum camphora, Diospyros japonica, Castanopsis eyrei, Quercus serrata, Rhus chinensis, Melia azedarach, Koelreuteria bipinnata,* and *Triadica cochinchinensis*	2, 4, 8, 16, and 24	Subtropical *P*: 1821 mm *T*: 16.7°C	Resistance	(Neighbourhood) tree species richness increases growth resistance of drought sensitive species in particular	Fichtner *et al*. (2020)
Drought	*Fagus sylvatica, Fraxinus excelsior, Tilia cordata, T. platyphyllos, Carpinus betulus, Acer pseudoplatanus, Quercus robur,* and *Q. petraea*	1 and 4	Temperate *P*: 590 mm *T*: 7.5°C	Resistance	Growth resistance of European beech increased with other species in the neighbourhood compared with con‐specific neighbourhoods. The positive effect was dependent on the species identity of the neighbours.	Mölder & Leuschner (2014)
Drought	*Fagus sylvatica, Fraxinus excelsior, Picea abies, Pinus sylvestris, Tilia cordata,* and *Quercus petraea*	1–6	Temperate *P*: 578 mm *T*: 9.4°C	Resistance	Tree species diversity did not affect drought‐induced mortality or resistance of soil functions. Soil function resistance depended on species identity.	Gottschall *et al*. (2023)
Drought	*Lithocarpus glaber, Schima superba, Daphniphyllum oldhamii, Quercus fabri, Cyclobalanopsis glauca, Castanea henryi, Castanopsis sclerophylla, Cyclobalanopsis myrsinifolia, Triadica sebifera, Liquidambar formosana, Castanopsis carlesii, Acer davidii, Sapindus saponaria, Nyssa sinensis, Quercus acutissima, Castanopsis fargesii, Choerospondias axillaris, Cinnamomum camphora, Diospyros japonica, Castanopsis eyrei, Quercus serrata, Rhus chinensis, Melia azedarach, Koelreuteria bipinnata,* and *Triadica cochinchinensis*	2, 4, 8, 16, and 24	Subtropical *P*: 1821 mm *T*: 16.7°C	Resistance	No general relief of water stress in species‐rich neighbourhoods; species identity and traits are playing a major role for drought resistance	Schnabel *et al*. (2024)
Drought	*Fagus sylvatica* and *Quercus robur*	1 and 2	Temperate (eight sites in Belgium) *P*: 800–1112 mm	Resistance	Increased drought stress for beech in mixture	Jacobs *et al*. (2021)
Drought	*Abies alba, Acer pseudoplatanus, Betula pendula, Carpinus betulus, Castanea sativa, Fagus sylvatica, Fraxinus excelsior, Ostrya carpinifolia, Picea abies, Pinus nigra, Pinus sylvestris, Quercus cerris, Quercus faginea, Quercus ilex, Quercus petraea,* and *Quercus robur*	1 up to 5	Gradient across Europe Boreal‐Mediterranean	Resistance	Drought stress may sometimes be reduced in mixed‐species forests, but this is not a general pattern, and even varies between sites for a given combination of species	Forrester *et al*. (2016)
Drought	21 different tree species from 5 biodiversity experiments: *Acer platanoides, Carpinus betulus, Quercus robur, Tilia cordata, Acer pseudoplatanus, Fagus sylvatica, Larix* × *eurolepis, Pseudotsuga menziesii, Quercus petraea, Acer saccharum, Betula papyrifera, Betula pendula, Quercus robur, Quercus rubra, Quercus ilex, Pinus pinaster, Acer monspessulanum, Arbutus unedo, Fraxinus ornus, Phillyrea latifolia, Pinus halepensis,* and *Quercus pubescens*	1–6	Temperate Atlantic to temperate continental	Resistance	The composition of species mixtures and thus species identity, rather than species richness, was identified as a driver of tree drought–mortality risk, suggesting that the effects can vary and may not always be positive	Decarsin *et al*. (2024)
Drought and pest	*Betula pendula* and *Pinus pinaster*	1 and 2	Temperate *P*: 893 mm *T*: 13.6°C	Resistance	Mixture with birch reduces pine processionary moth infestation but not pine stem borer attack under drought	Poeydebat *et al*. (2021)
Drought and pest	*Quercus robur, Q. pyrenaica, Q. ilex, Betula pendula,* and *Pinus pinaster*	1–4	Temperate *P*: 893 mm *T*: 13.6°C	Resistance	Tree species richness lowered leaf miner abundance, leaf chewer damage, and oak powdery mildew infection, but the effect on leaf miners was smaller under drought. Tree neighbour identity plays also a role.	Field *et al*. (2020)
Drought and pest (bark beetles)	*Acer platanoides, Acer saccharum, Betula papyrifera, Betula pendula, Larix decidua, Larix laricina, Picea abies, Picea pungens, Pinus strobus, Pinus sylvestris, Quercus robur,* and *Quercus rubra*	1, 2, 4, 6, and 12	Temperate *P*: 832 mm *T*: 11.8°C	Resistance	Increasing tree diversity reduces the risk of bark beetle infestation for species prone to high infestation rate but increases the risk for less preferred species due to spill over from the preferred hosts	Berthelot *et al*. (2021)
Heat	*Castanea sativa, Ostrya carpinifolia, Quercus cerris, Quercus ilex,* and *Quercus petraea*	1–4	Mediterranean *P*: 825 mm *T*: 14.2°C	Resistance and recovery	Growth resistance and recovery increased with tree species diversity	Iacopetti *et al*. (2023)

Literature was retrieved from the Web of Science (Core Collection) using the search string (all fields): (‘Tree diversity’ OR ‘Tree species mixture’ OR ‘Tree species diversity’) AND (resistance OR resilience OR recovery) AND (drought OR heat). We omitted pure modelling studies and glasshouse experiments in pots. Only studies that assessed how biodiversity affects tree resistance, resilience, or recovery from heat or drought events were included. Diversity gradients: number of tree species. Blue‐coloured rows indicate overall positive effects of tree diversity, and orange colours show negative impacts. Noncoloured rows indicate no clear or mixed effects.

The management for – or and the prediction of – drought stress mitigation thus requires consideration of the structural and physiological traits of the species in a mixture and their trait complementarity, together with the environmental conditions (Schnabel *et al*., 2024). Table 3 illustrates trait complementarities that may enhance drought tolerance while (partially) also increasing the temporal stability of functioning, thereby reducing the difference between wet and dry years. This temporally stabilising effect may occur, especially when both drought‐sensitive trees, which may be more efficient in resource use under moist conditions, and drought‐tolerant species are mixed. However, interspecific neighbourhood interactions could, although primarily beneficial, trigger mortality if competition becomes imbalanced under stress, leading to concerted C starvation and hydraulic failure during intense drought in the less competitive species (Hajek *et al*., 2022). In a tree diversity experiment, drought‐tolerant species with a high hydraulic safety limit, such as *Quercus rubra* and *Acer platanoides*, reduced the survival probability of less drought‐tolerant co‐occurring species (e.g. *Larix decidua, Betula pendula* and *Betula papyrifera*), most likely as a result of continuing water extraction beyond the cavitation threshold of the less tolerant species (Hajek *et al*., 2022). Thus, not only (and maybe not even primarily) species richness but also the combination of tree species with compatible and complementary traits should be the focus of CSF.

**Table 3:** Tree species combination with complementary traits that may enhance drought tolerance.

Species combination	Complementary traits	Effect on drought tolerance	References
Species with different mycorrhizal types	Ectomycorrhizal (ECM) + Arbuscular mycorrhizal (AM) species	Enhanced resistance and resilience – Diverse mycorrhizal networks improve water and nutrient acquisition under drought	Sachsenmaier *et al*. (2024)
Drought‐tolerant + Drought‐sensitive species	Different water use strategies; contrasting hydraulic leaf traits (e.g. turgor loss point). Different stomatal control and thus drought impact on photosynthesis	Mixed effects – Benefits during moderate drought, but competition may reduce resistance during and resilience after extreme drought; Species respond differently in wet vs dry years, providing temporal stability	Hajek *et al*. (2022); Schnabel *et al*. (2024)
Deep‐rooted + Shallow‐rooted species	Vertical niche differentiation in water uptake and potential for hydraulic redistribution	Enhanced water resource utilisation – Reduced competition for water at different soil depths, facilitation via hydraulic redistribution	Grossiord (2020)

Species diversity in forests can also significantly impact the dynamics of forest pathogens. This is particularly important since pest and pathogen disturbances will increase in a warmer world with higher precipitation variability (Ayres & MaJ, 2000; Hartmann *et al*., 2025b). Guyot *et al*. (2016) observed that increasing tree diversity reduced damage by nongeneralist pests in mature forests across a gradient from the Mediterranean to the boreal zone in Europe. However, some studies suggest that pure stands exhibit lower pathogen pressure in specific contexts than mixed stands, as these may be more attractive for generalist herbivores (Koricheva *et al*., 2006; Vehviläinen *et al*., 2007). These authors also suggested that species identity, rather than richness, affected the damage caused by pests and diseases, pointing to the critical role of phytochemical diversity in mediating plant–enemy interactions (Fahey *et al*., 2025). Understanding the effect of species diversity on forest pathogens and pathogen damage also requires accounting for the environmental context (Ratcliffe *et al*., 2017), soil feedbacks (Liu & He, 2019), and the occurrence of both species‐specific and generalist pathogens. Especially when pests or pathogens target a specific tree species, the presence of other tree species in the ecosystem can reduce the overall impact or prevent the complete loss of the forest cover. However, increased pathogen pressure on nonpreferred tree species also needs to be accounted for (see Table 2 and Berthelot *et al*., 2021). The defence capability against pests and pathogens depends on the impact of heat and drought on plant metabolism (Section II.2) and on pest population dynamics. Reducing drought stress in suitable species mixtures should lead to higher C and N availability for allocation to defence compounds (Anderegg *et al*., 2015; Gessler *et al*., 2017), thus mitigating the damage potential.

With respect to forest fires, there is a negative relationship between tree species diversity (specifically, the proportion of broadleaved trees in conifer‐dominated stands) and fire risk. Thus, increasing the proportion of broadleaf species in conifer forests leads to improved fire resistance and reduced fire damage (Park *et al*., 2024). This is because broadleaved trees often have a higher moisture content and lower flammability, which reduces the spread and intensity of fires. Tree flammability traits may help to select particular species with low flammability (Popović *et al*., 2021). In addition, high tree diversity shows a temperature buffering effect (Schnabel *et al*., 2025) that could potentially reduce fire risk by maintaining cooler, more humid conditions within diverse forests.

#### Implications for climate‐smart forestry

**Fig. 5:**
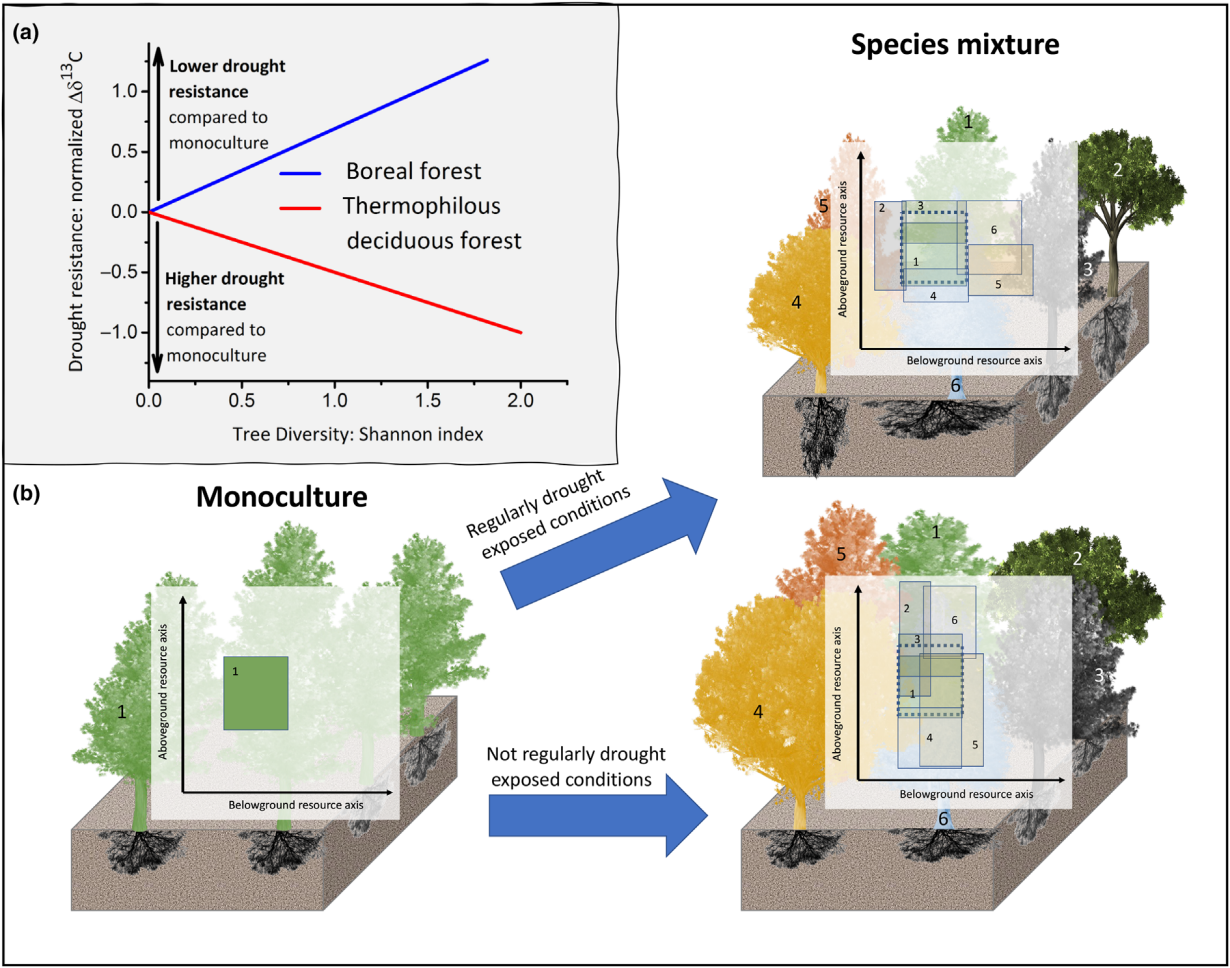
Potential impacts of long‐term water‐related climatic history on below‐ and aboveground structure and resource partitioning in species mixtures compared with monocultures. (a) The relationship (regression lines) between tree diversity (Shannon index) and the resistance to drought for boreal forests (Grossiord *et al*., 2014) and thermophilous deciduous forests (Grossiord *et al*., 2013). Resistance to drought was calculated from tree ring carbon isotope composition in a dry compared with a wet year and normalised to the monocultures of each forest type. In boreal forests, drought resistance decreases with increasing tree diversity, whereas it increases in thermophilous forests. (b) Effects of the long‐term water‐related climatic history on the below‐ vs aboveground resource‐space exploitation in mixtures vs monocultures. Different colours and numbers of trees and the areas in the inserted *x*‐*y* graphs indicate different species. The dotted areas on the right‐hand side inserts indicate the monoculture's resource space.

The trees' functional trait diversity must be put into the context of the site's water‐related climatic history as well as edaphic and nutrient‐related features, and requires careful evaluation of the resource demands of the species in a mixture. Intra‐ and interspecific interactions between trees growing in different regions may be associated with varying expressions of trait complementarity in the context of drought resistance, and species identity itself may profoundly influence diversity effects (Sebald *et al*., 2021). In areas with no regular history of heat or drought events, complementarity and facilitation in mixed stands composed of local tree species may be shaped by the prevailing environmental conditions to serve purposes other than drought resistance. For example, in forests where water (and nutrient) availability usually is not a limiting factor and thus competition for light is prevalent (Forrester, 2014), trees growing in mixed stands may mainly benefit from a better exploration of aboveground space than monocultures (Fig. 5b). However, this may result in a lower root‐to‐shoot ratio and therefore cause hydraulic restrictions under drought. In fact, increasing tree diversity was found to reduce forest resistance to drought in boreal forests with no regular drought exposure, while it was beneficial in regularly drought‐prone thermophilous and temperate forests, most likely due to increased belowground complementarity (Fig. 5a, Grossiord *et al*., 2013; Grossiord *et al*., 2014). Hence, when we assume that ecosystems are regularly exposed to drought, water (and soil nutrients; see Gessler *et al*., 2017) becomes limiting, and species mixtures will increase belowground resource partitioning, thus increasing the resource space along the belowground resource axis, compared with monocultures (Fig. 5b). This will be achieved primarily by complementary rooting structures, which are suited to exploit soil resources optimally, leading to increased drought resistance (thermophilous forest in Fig. 5a). This is due to a high water‐absorbing root surface compared with a relatively small transpiring leaf surface. By contrast, when the system is not (or only rarely) water‐limited, aboveground resource partitioning through improved space occupation will increase the resource space mainly along the aboveground resource axis to optimally intercept light. In the latter case, drought events, as occurring with climate change also more frequently in previously not drought‐affected areas, may cause increased drought stress due to unfavourable root‐to‐shoot ratios. Such differences in expanding the belowground vs aboveground resource space in mixtures compared with monocultures, depending on the environmental context and the climatic history, can explain that tree species diversity may reduce drought resistance in some regions and forest ecosystems, but not in others.

In addition, belowground facilitation via hydraulic lift and redistribution may occur in mixed forests (Kitajima *et al*., 2013), provided that there is complementarity between deep‐ and shallow‐rooting species (see Section IV). In the Hainich National Park in Germany, where Grossiord *et al*. (2014) observed an increase in drought resistance with increasing tree diversity, fine‐root abundance and recovery after disturbance were also positively affected by tree diversity (Meinen *et al*., 2009), pointing to the importance of belowground resource acquisition. These findings have implications for CSF: when using the local species pool in regions that are usually not drought‐exposed to establish forests with high tree species diversity, belowground trait complementarity needs particular care. In boreal forests, where adverse effects of biodiversity seem to be most critical in this respect (Fig. 5a), *Populus tremuloides* and *Pinus banksiana* mixtures showed increased belowground complementarity compared with their monocultures (Ma *et al*., 2019), while mixtures of *B. pendula*, *P. sylvestris* and *P. abies* did not.

Moreover, in generally favourable environments, tree diversity may additionally cause an increase in overall competitive intensity through enhanced productivity, leading to higher tree mortality during extreme events (Searle *et al*., 2022). As a consequence, CSF should include competition management and reduction (e.g. see Pretzsch, 2022) to maintain the ecological carrying capacity (Del Monte‐Luna *et al*., 2004) also under adverse conditions.

In general, our considerations underscore that the local environment, the most limiting resources, and the history of prevailing climatic conditions may determine whether below‐ or aboveground complementarity is expressed in tree species mixtures. However, considering species identity and selecting complementary above‐ and belowground traits in mixtures allows management to modify the degree of above‐ vs belowground complementarity and improve drought sensitivity.

##### Key points for climate‐smart forest management

Mixing different tree species can enhance resilience and resistance to drought, but may also have adverse effects. Tree species identity, trait complementarity, and the environmental context must be taken into account. Additionally, the portfolio effect of mixtures reduces the likelihood of stand‐replacing disturbances.

### 3. Stand density

Under favourable site conditions, that is adequate water and nutrient availability, high stand density typically leads to a higher leaf area index (Le Dantec *et al*., 2000) and, consequently, greater water demand due to increased transpiring leaf area. However, maximum leaf area is ultimately constrained by site resources, particularly soil fertility, water availability, and climatic conditions (Cowling & Field, 2003). In resource‐limited environments, dense stands may not achieve a high leaf area index, and site quality becomes the primary determinant of canopy development. However, stand density reductions are generally recommended as they at least temporarily reduce leaf area and, hence, stand‐level transpiration (del Campo *et al*., 2022). Yet, since canopy structure strongly affects forest microclimate (De Frenne *et al*., 2021), stand density reductions that lead to less dense, open forests result in higher temperatures within the stand, down to the forest floor. Thus, the forest interior climate may be less buffered during heatwaves.

#### Ways to attenuate the physiological damage mechanisms

**Fig. 6:**
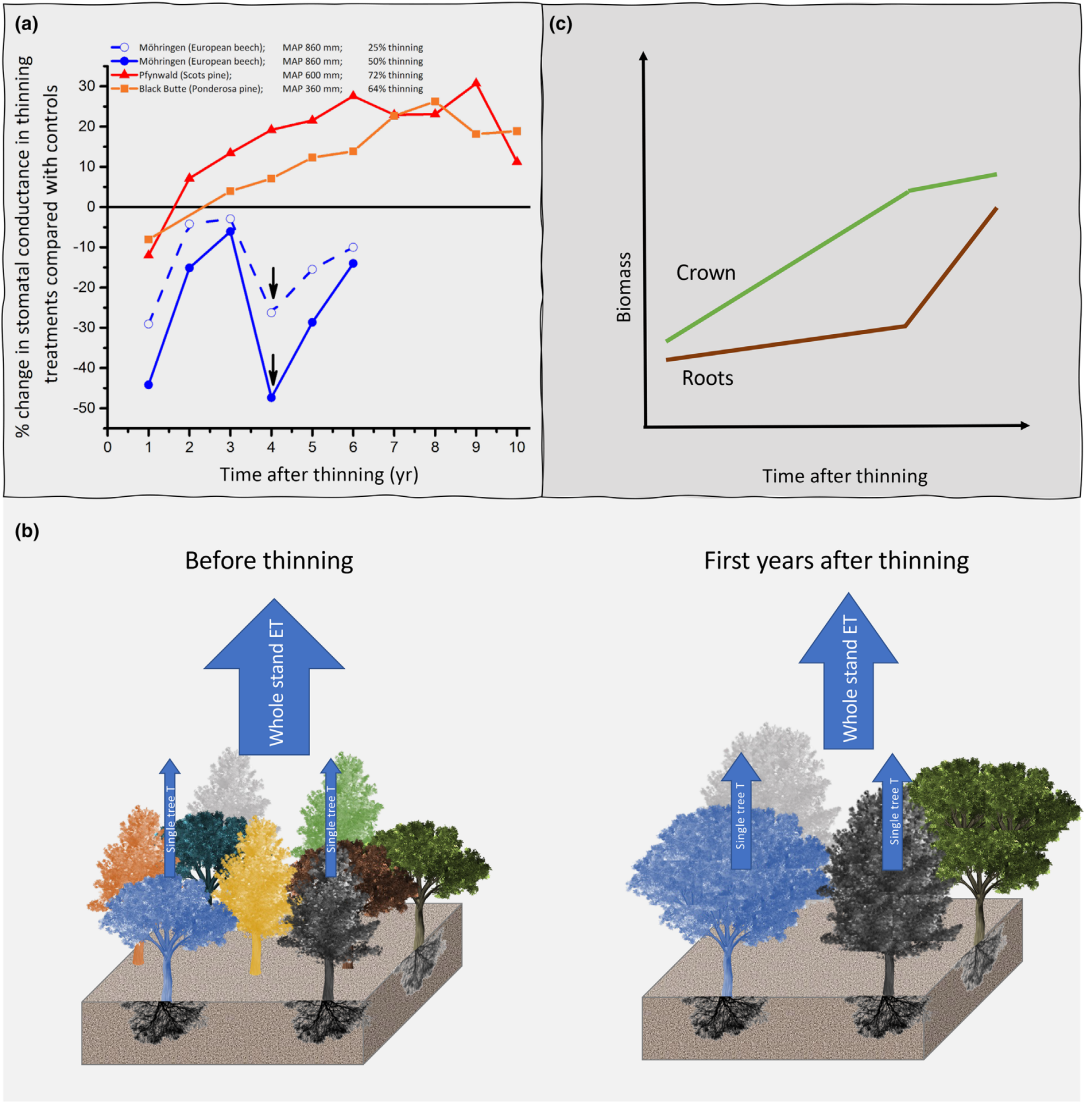
Effects of thinning on tree and stand water use in the first years after the intervention. In (a), the effect of thinning on the stomatal conductance of the remaining trees is shown for up to 10 yr after the intervention. Data from Pfynwald (Giuggiola *et al*., 2016) and Black Butte (McDowell *et al*., 2003) are derived from tree ring carbon and oxygen isotope values, and data from Möhringen are calculated using xylem flow measurements. From Möhringen, data for the first year after thinning (and the methodology) are published by Keitel *et al*. (2003), and data from the other years are unpublished. Vertical arrows in (a) show the extremely hot and dry year 2003. MAP, mean annual precipitation. In (b), we propose that in usually not water‐limited stands (such as Möhringen), thinning mainly releases aboveground competition and biomass is primarily allocated to the crown to occupy the newly available space. In these cases, biomass allocation to roots may only follow later (c) to compensate for the increased water demand of a larger crown. Even though whole‐ecosystem water use (whole stand ET in (b)) may decrease, the remaining trees might become more drought sensitive for several years after thinning as a result of the larger crown and thus higher tree‐level water use (single tree T) but an unchanged root system and hence a higher shoot‐to‐root ratio. Different colours for trees in (b) indicate different individuals (and are unrelated to any species).

Density reduction across many forest ecosystem types has been shown to increase drought resistance (e.g. Giuggiola *et al*., 2013; Bottero *et al*., 2017; Bradford *et al*., 2021) by improving water availability for the remaining trees (Giuggiola *et al*., 2016; Sohn *et al*., 2016). Increased water availability reduces the risk of hydraulic failure, thereby decreasing mortality and enhancing growth. In these cases, we can also assume that reduced belowground resource competition improved nutrient supply, positively affecting general metabolic function and defence (Gessler *et al*., 2017). Although density reduction can ameliorate drought impacts on growth, this is not always the case (Castagneri *et al*., 2022). From 27 publications that assessed the effect of stand thinning on soil water availability and tree water relations (Table 2), only 14 showed an overall positive effect during drought events, whereas five reported primarily negative impacts on tree water relations. In the remaining studies, there are either no clear effects or mixed effects corroborating the results of a recent meta‐analysis (Willig *et al*., 2025). Increased drought stress has, for example, been observed in *F. sylvatica* forests on dry‐warm exposures in the first years after thinning (Fig. 6a; site Möhringen; Simon *et al*., 2017). This stress has been attributed to high soil evaporation and understorey transpiration due to transient canopy opening, leaving less water for the overstorey trees and leading to stomatal closure (Gessler *et al*., 2004).

#### Implications for climate‐smart forestry

The inconsistent effects of thinning on soil water availability and tree water relations during drought (Table 4) suggest that the local environmental context, such as climate and soil characteristics, play a critical role. Thinning results in reduced stand water use, and canopy opening induces aboveground competition release and, thus, substantial investment in aboveground biomass to occupy the freed space (e.g. Bayer & Pretzsch, 2017). A potential transient reduction in drought resistance and resilience may occur if the root‐to‐shoot ratio becomes less favourable, leading to disproportionate increases in aboveground relative to belowground biomass, resulting in a structural overshoot of tree growth (Jump *et al*., 2017). There are indications that the long‐term water availability at a given site affects the risk of increased drought susceptibility in the years after thinning: at the xeric sites Pfynwald (Switzerland) and Black Butte (United States; Fig. 6a), stomatal conductance (after decreasing in year one after thinning) increased in the trees of the thinned compared with the control stands over at least 10 yr, indicating increased water availability. At the comparably moist stand in Möhringen (Germany), stomatal conductance did not reach the values of the unthinned controls in the first 3 yr and dropped again in Year 4, which was in response to the extremely hot and dry year 2003 (vertical arrows in Fig. 6a), strongly impacting large parts of Europe (Ciais *et al*., 2005). This indicates higher drought stress in the thinning treatments than in the controls, and the effect was more pronounced in the higher‐intensity (50%) thinning treatment. After 2003, stomatal conductance converged with the control values.

**Table 4:** Effects of stand density regulation on soil water availability and tree water relations during drought.

Thinning treatment	Species	Time between thinning and drought (yr)	Climatic region and climatic conditions	Impact on soil water availability	Impact on tree water relations	References
Basal area reduction to 29% (repeated twice) of controls	*Cedrus atlantica*	13 after the first thinning, 2 yr after the second	Mediterranean *P*: 1667 mm *T*: 9.6°C	nd	Increased resistance and improved the postdrought recovery for up to a decade	Guillemot *et al*. (2015)
Basal area reduction to between 20 and 84%	*Cryptomeria japonica*	2	Temperate: *P*: 3200 mm *T*: 12.1°C	Higher soil water availability during a drought event	nd	Weng *et al*. (2007)
Basal area reduction to between 50 and 25%	*Fagus sylvatica*	4	Temperate *P*: 860 *T*: 6.6	nd	Reduced stomatal conductance; more pronounced in the 50% thinning	Simon *et al*. (2017); Keitel *et al*. (2003); Fig. 6
Stem number reduction (repeated) to between 60 and 95%	*Picea abies*	6–7 and 30 after the first treatment	Temperate *P*: 790 and 780 mm *T*: 7.9 and 7.5°C	nd	6–7 yr after thinning, stomatal conductance and growth are higher during drought; 30 yr after the initial treatment, growth resistance was lower, but growth recovery faster.	Sohn *et al*. (2013)
Basal area reduction to 57 and 33%	*Picea abies*	na	Temperate *P*: 778 mm; *T*: 7.9°C	Higher soil water availability	Decreased stand water use, which decreases after 3 yr due to increased single tree transpiration. Reduced fine‐root biomass but no change in leaf area‐to‐root biomass ratio. High thinning might increase competition for water with understorey vegetation.	Gebhardt *et al*. (2014)
Canopy competition reduction from 54 to 0%	*Picea rubens*	na	Humid‐continental *P*: 1064 mm *T*: 6.5°C		Strong increase in *T*_air_ and VPD. Reduction in midday water potential in the weeks after thinning and reduction in photosynthetic efficiency for the whole growing season (thinning shock).	French *et al*. (2023)
Basal area reduction to 67 and 50%	*Pinus halepensis*	Regular drought	Semiarid Mediterranean *P*: 280 mm *T*: 18.2°C	N.D	Increased stomatal conductance, assimilation, and growth	Moreno‐Gutiérrez *et al*. (2011)
Basal area reduction to between 23 and 67%	*Pinus halepensis*	6	Mediterranean *P*: 477 mm *T*: 14.1°C	nd	Higher water use and WUE than control plots due to an increase in tree growth	Fernandes *et al*. (2016)
Stem number reduction by up to 96%	*Pinus contorta*	5	Boreal	Soil water availability low in the heavily thinned stand due to soil evaporation	Improved water use efficiency, drought tolerance, and drought recovery by decreasing stand density and improving carbon storage	Ellis *et al*. (2024)
Basal area reduction to between 32 and 57%	*Pinus halepensis*	Regular summer drought	Mediterranean *P*: 550 mm *T*: 20°C	nd	Lower predawn water potential and mortality higher growth; effects increased with time	Calev *et al*. (2016)
Basal area reduction to 75%	*Pinus nigra*	10	Temperate *P*: 663 *T*: 11.3	nd	No change in (intrinsic) water use efficiency; increase in temperature and irradiance, decrease in RH	Martín‐Benito *et al*. (2010)
Increase in spacing between trees	*Pinus nigra*	Regular summer water deficit	Mediterranean *P*: 1276 mm *T*: 12.1	Higher soil water availability	Higher stomatal conductance and leaf level photosynthesis, less negative midday water potential	Deligöz *et al*. (2019)
Creation of irregular tree groups, increasing overall interspace, significant reduction in smaller trees.	*Pinus ponderosa*	1–3	Dry‐Temperate (Semiarid) *P*: 560 mm; Summer droughts *T*: 6.6°C	Soil water availability during drought was increased with reduced stand‐level basal area, canopy cover and tree density, reduced with shorter trees	nd	Belmonte *et al*. (2022)
Basal area reduction to between 39 and 18%	*Pinus ponderosa*	7	Dry‐temperate *P*: 340 mm *T*: 8.2°C	nd	Increased stomatal conductance, less negative tree water potential, and higher growth	McDowell *et al*. (2003); Fig. 6
Basal area reduction to between 15 and 75% (with four repeated treatments every 10 yr)	*Pinus ponderosa*	Regular summer drought	Dry‐temperate *P*: 564 mm *T*: 6°C	Higher soil water availability	Generally increased growth, photosynthesis and stomatal conductance, leaf area to sapwood area. Increase in leaf area to sapwood area increases sensitivity to drought.	McDowell *et al*. (2006)
Basal area reductions (repeated (*c*. 10 yr‐intervals)) to between 20 and 60%	*Pinus resinosa*	4 and 31 after the first thinning	Temperate *P*: 766 mm *T*: 2.8°C	nd	Higher tree resistance and resilience towards the first drought (after 4 yr). Lower resistance and resilience towards the second drought.	D'Amato *et al*. (2013)
Basal area reduction to 87%	*Pinus strobus*	1	Temperate *P*: 832 mm *T*: 8.1°C	Higher soil water availability	Lower stand water use but higher single tree transpiration in the drought year	Skubel *et al*. (2017)
Basal area reduction to 66%	*Pinus sylvestris*	Regular summer droughts	Temperate *P*: 600 *T*: 10.1	Higher soil water availability	Increased stomata conductance	Giuggiola *et al*. (2016); Fig. 6
Basal area reduction (repeated) to between 40 and 75%	*Pinus sylvestris*	Several droughts between 2 and 13	Temperate Several sites in Germany *P*: 500–650 mm *T*: 7.5–10.3°C	nd	Growth recovery was higher shortly after thinning and declined with time	Sohn *et al*. (2016)
Basal area reduction to 76%	*Pinus sylvestris, Picea abies*	2	Boreal *P*: 527 mm *T*: 5.5°C		Up to seven times higher water use (transpiration) in the thinned plot during drought	Lagergren *et al*. (2008)
Basal area reduction to 18%	*Ponderosa pine*	1	Dry‐temperate *P*: 442 mm *T*: 6.7°C	1 yr after thinning soil water availability was higher, but there were no differences in year 2	Reduced stomatal conductance and whole‐tree conductance in smaller (thinning shock) but not in larger trees during drought	Simonin *et al*. (2006)
Basal area reduction to 33%	*Pseudotsuga menziesii*	14	Temperate *P*: 1160 mm, with 125 mm from June to September; *T*: 8.9°C	Higher soil water availability	Changes in microclimate with lower RH and higher leaf temperature	Brooks & Mitchell (2011)
Stand density gradients (from 20 to 200 trees ha^‐1^)	*Quercus ilex*	Regular summer drought	Mediterranean Dry site *P*: 506 mm *T*: 16.2°C	nd	No clear effect in photosynthesis and water potential	Moreno & Cubera (2008)
Stand density gradients (from 20 to 200 trees ha^‐1^)	*Quercus ilex*	Regular summer drought	Mediterranean Wet site *P*: 816 mm *T*: 14.7°C	Higher soil water availability	Increase in photosynthesis and sap flow, less negative water potential	Moreno & Cubera (2008)
Basal area reduction to 70%	*Quercus ilex*	Same year	Mediterranean *P*: 914 mm *T*: 13.1°C	nd	More negative predawn leaf water potential in the 2 yr following the treatment	Rodríguez‐Calcerrada *et al*. (2011)
Basal area reduction to 70%	*Quercus ilex*	27% rainfall exclusion experiment	Mediterranean *P*: 910 mm *T*: 13.2°C		Reduced sensitivity towards drought; increased primary productivity; decreased stand transpiration and mortality	Gavinet *et al*. (2019)
Basal area reduction to 65%	*Quercus petraea*	1	Temperate *P*: 744 mm; *T*: 9.2°C	Higher soil water availability	Less negative tree water potential and higher growth	Bréda *et al*. (1995)

Literature was retrieved from the Web of Science (Core Collection) using the search string (all fields): Forest* AND thinning AND Drought AND (‘water potential’ OR ‘water use’ OR Transpiration OR ‘stomatal conductance’ OR ‘soil water’). We omitted pure modelling studies and assessments of very young commercial plantations. Studies included had to assess thinning effects during natural or experimentally induced drought events, with two exceptions: Gebhardt *et al*. (2014) assessed thinning impact over several years with no reported drought. However, they evaluated the root biomass‐to‐leaf area ratio, an important indicator for above‐ and belowground trait coordination. French *et al*. (2023) investigated short‐term responses in the weeks to months after thinning. Blue‐coloured rows indicate overall positive effects of thinning, and orange colours show negative impacts. Noncoloured rows indicate no clear or mixed effects. na, not applicable; nd, not determined; RH, relative air humidity.

In Fig. 6(b), we propose a mechanism for the observations reviewed above. In a normally nonwater‐limited stand (such as Möhringen), thinning mainly releases aboveground competition, and biomass is primarily allocated to the crown to occupy the newly available space. If water and nutrients are not limiting, allocation to roots may follow only later (Fig. 6c) to compensate for the increased water demand of a larger crown. Such a temporal sequence between above‐ and belowground biomass allocation was also observed in a long‐term irrigation experiment. After drought release upon irrigation, leaf biomass and crown size expanded first, and only several years later did root biomass increase (Bose *et al*., 2022). Even though whole stand ET may decrease (see Fig. 6b and Keitel *et al*., 2003), the remaining trees may become more drought‐sensitive for several years after thinning as a result of a larger crown and thus higher tree‐level water use (single tree T in Fig. 6b) but an unchanged root system and hence a higher shoot‐to‐root ratio. Therefore, when a severe drought occurs in the first year(s) after treatment, a higher cavitation risk may result from the overbuilt crown. In regularly drought‐exposed ecosystems (such as Pfynwald and Black Butte), such processes may not occur, as under these conditions, thinning releases trees from water restriction rather than from aboveground competition (Giuggiola *et al*., 2016).

Thus, CSF needs to take precautions, particularly in mesic and fertile locations where trees could become more susceptible to drought and drought‐induced damage for some time after a thinning intervention. In particular, moderate thinning (Fig. 6: 25 vs 50% thinning for Möhringen) may mitigate the negative impacts. However, this conceptual model requires further testing with different tree species and thinning regimes under varying soil and climatic conditions to provide more detailed information for forest management practice.

Thinning can increase soil and within‐stand air temperature, with various consequences for the understorey and belowground processes. Canopy opening by thinning affects soil water availability in the upper soil layer and transpiration of understorey trees (Fotelli *et al*., 2003). Depending on the prevailing environmental conditions the thinned stand is exposed to, this can have positive (e.g. under cool and moist conditions) or adverse (under warm and dry conditions) effects on tree seedling growth, which is also related to competition between tree regeneration and other understorey plant species (Fotelli *et al*., 2004). Since thinning is also a means to stimulate tree regeneration, CSF needs to account for potential negative effects on tree seedlings and saplings. Increased soil temperatures not only affect vegetation but also the activity of soil microbial communities and may induce carbon loss from the soil via increased heterotrophic soil respiration (Schindlbacher *et al*., 2009). However, other studies have shown that additional carbon loss from the soil via respiration is negligible (Tang *et al*., 2005; Olajuyigbe *et al*., 2012; Pang *et al*., 2013), and the effect may depend on tree species and thinning intensity (Zhang *et al*., 2018).

A critical question for CSF is how long a thinning intervention can reduce stand water use and thus make a stand more drought‐resilient. The expansion of the tree crown and leaf area of the remaining trees over time will increase stand transpiration, which eventually will reach and possibly even exceed preintervention levels. It has been shown that the duration of thinning effects on the stand water balance depends directly on thinning intensity (Sohn *et al*., 2016). While low‐intensity thinning might lead to the recovery of stand water use within a few years only (Bréda *et al*., 1995; Lagergren *et al*., 2008), heavy interventions (e.g. reducing stand basal area by > 70%) can, in particular cases, have decade‐lasting effects on the water balance (Giuggiola *et al*., 2016). However, this is rather the exception than the rule, as thinning effects generally vanish after several years (Elkin *et al*., 2015). Since heavy thinning may also induce structural overshoot, repeated low‐intensity thinning appears to be the most promising approach. However, this comes with considerable costs for forest operations.

In conclusion, reducing stand density can improve the drought response of individual trees and whole stands, but site‐specific conditions such as climate and competition regimes (light vs water) may strongly modulate these responses, sometimes leading to increased or reduced drought stress with lower stand density (Table 4; Moreno & Cubera, 2008; Rodríguez‐Calcerrada *et al*., 2011). The potential advantages of stand density reductions may be best achieved during the thinning phase of forest stands, while in later developmental phases, they have to be approached with caution (Willig *et al*., 2025). In older and taller forests, where stand density reductions are likely not considered as thinning but carried out in the form of selection harvests and for regeneration purposes, new approaches need to be developed for vulnerable stands to avoid strong heat, high VPD, and also wind exposure of canopy trees. Moreover, to evaluate whether thinning could lead to structural overshoot, more information is needed on how trees partition biomass between above‐ and belowground compartments after thinning under different water availability.

##### Key points for climate‐smart forest management

Thinning is a suitable tool for reducing water use in stands and thereby decreasing drought stress. This has been mainly shown in dry areas. Intensive thinning should be considered cautiously in usually well‐watered (and well‐nutrient supplied) stands, as there might be a risk of structural overshoot aboveground, leading to increased drought susceptibility in the first years following intervention. Still, more information is necessary to determine whether this is a species‐specific response and a stand‐specific effect. Moderate and repeated thinning operations are suggested as mid‐ to long‐term measures to increase drought resistance and resilience.

### 4. Stand structure, tree height and age distribution, and production times

Along with species composition and tree density, forest management affects stand structure, tree height, and age distribution. These stand properties can be targeted to mitigate the adverse effects of climate change.

#### Ways to attenuate the physiological damage mechanisms

Stands with larger and taller trees are often more vulnerable to drought (Fajardo *et al*., 2019; McGregor *et al*., 2021; Fernández‐de‐Uña *et al*., 2023) due to hydraulic constraints and, thus, stress to the hydraulic system under drought (see Section II.1, Bennett *et al*., 2015; Grote *et al*., 2016). In addition, tall trees are exposed to greater damage from wind, temperature, VPD, and lightning (McDowell *et al*., 2020). For some species, large trees are also more vulnerable to bark beetle infestations (Netherer & Nopp‐Mayr, 2005; Pfeifer *et al*., 2011), mainly because they have a thicker phloem as a food source and a greater area for colonisation (Kärvemo *et al*., 2023). Aiming for smaller‐sized trees has, thus, been suggested as a management strategy to be adopted in the face of increasing droughts (Bottero *et al*., 2021), allowing a shift in the partitioning of growth in favour of tree sizes less vulnerable to drought (Bose *et al*., 2021). Managing for smaller tree sizes can be achieved by reducing production time and increasing the frequency of interventions. Note that we here use ‘production time(s)’ instead of the often synonymous used term ‘rotation times’. The latter is associated with even‐aged stands harvested by clearfelling (therefore rotations), whereas the former can also be used for individual trees.

When the target diameters of trees are to be maintained despite shorter production cycles, individual‐tree growth must be accelerated through increased frequencies and/or intensities of thinning interventions, resulting in lower height‐to‐diameter ratios of crop trees (but see also Section III.3 for the potentially adverse effects of high thinning intensity).

The height‐to‐diameter ratio, crucial for hydraulic safety (see Section II.1), can thus be efficiently controlled through thinning practices (Slodicak & Novak, 2006). Low ratios may help increase stand drought resistance by preventing hydraulic failure during drought periods. At the same time, lower height‐to‐diameter ratios increase the structural stability of individual trees and stands, making them less susceptible to other disturbances like windthrow and snow or ice damage (e.g. Suliman & Ledermann, 2025).

The age class distribution can also affect the coupling of forest carbon and water relations, thus influencing drought vulnerability and carbon sequestration potential. Even‐aged conifer forests may have a decreasing water use efficiency from young to mature stands (Tang *et al*., 2017; Arango Ruda & Arain, 2025) even though for single trees, a positive relationship between (intrinsic) water use efficiency and tree height has been observed (McDowell *et al*., 2011b). Uneven‐aged forests sustain high water use efficiency over extended periods (Tang *et al*., 2017), making them less vulnerable to drought. Tree age and thus height in uneven‐aged forests has, however, been reported to affect drought responses in different ways (negative effect of tree age and height: Floyd *et al*. (2009), Martínez‐Vilalta *et al*. (2012); positive effect: Cavin & Jump (2017)), and the effect may be mediated over the years by changes in the carbon allocation between shoots and roots or in the hydraulic system related to tree size (Ryan *et al*., 2006). However, the concurrent presence of different age classes and structures, which are differently affected by drought in uneven‐aged forests, may buffer drought responses at the stand level (Teets *et al*., 2018; Bottero *et al*., 2021). Structural diversity resulting from diverse age class distributions can also lead to increased niche complementarity (Dănescu *et al*., 2016; Forrester & Bauhus, 2016). A larger variability in rooting depths across age classes may result in better exploitation of soil water resources (as species diversity does; see Fig. 5), and canopy stratification can improve light interception. Another example that age diversity can convey functional trait diversity is stomatal sensitivity to VPD that changes with tree age within a given species (e.g. Ewers *et al*., 2005), leading to a broader whole‐ecosystem range within the isohydric–anisohydric continuum. Although observed in case studies, it is unclear whether the effects of tree size diversity on drought tolerance can be generalised (Dănescu *et al*., 2018; Pretzsch *et al*., 2018), and thus, additional research is needed.

#### Implications for climate‐smart forestry

The shortening of production time (i.e. time from establishment to the final felling of a tree) is a management adaptation action suggested to face increasing drought risks (Spittlehouse & Stewart, 2004) by reducing the time of exposure to potential drought events and the drought vulnerability associated with ageing trees as they grow taller. Additionally, shorter production time may allow for faster and more proactive adjustments of species or provenances suitable for the new climate (Schelhaas *et al*., 2015), hence reducing the potential tree species bottleneck (see Section III.1). Additionally, the more frequent interventions required for this goal provide more opportunities to maintain tree vitality and a diverse species composition, respond to new developments and unforeseen problems, and thus keep lag times in management short, thereby maintaining high resilience and adaptive capacity (Puettmann & Bauhus, 2023). However, these measures may also have adverse effects (Zimová *et al*., 2020). A shorter production time will negatively impact total forest carbon storage and the abundance of large trees (Zimová *et al*., 2020), which are often considered essential elements for biodiversity on the one hand and for valuable timber on the other. With shorter production time, soil erosion and nutrient depletion may increase (Worrell & Hampson, 1997), and the soil microbial community may shift towards microbes that are harmful to the trees (Bose *et al*., 2023), causing negative feedback on drought resistance and forest functioning in general.

For biodiversity conservation, shorter production times are viewed critically and have a perceived low acceptance, as many forest values and functions are associated with large trees and long‐term habitats and habitat traditions (Himes *et al*., 2023). To address this problem, patches of old forest and habitat trees may need to be retained in parts of the landscape (e.g. shaded slopes and moist gullies), where they experience less heat and drought stress.

##### Key points for climate‐smart forest management

In areas at risk of recurring intensive drought periods, shorter trees with lower height‐to‐diameter ratios can be the management aim to decrease the risk of hydraulic failure. This can be achieved by selective felling or reduced production time and supported by increased structural diversity with different age classes. Negative aspects such as biodiversity loss and lower carbon storage must be accounted for, and on a landscape level, patches of old forests in suitable local habitats can be used to mitigate some of the trade‐offs.

### 5. Nutrients and soil pH

Nutrient addition is rarely practised in Central European forests (except for liming aimed to counteract soil acidification) but is a common practice in plantations in the United States, France, Spain, and Portugal, and (less common) in northern Europe (Smethurst, 2010). In Central Europe, excess N input via atmospheric deposition can have detrimental effects on tree and forest growth (Etzold *et al*., 2020). Such growth repressions are due to nutrient imbalances and soil acidification (Du *et al*., 2019), and to a reduction in root biomass and mycorrhizal abundance and diversity (Lilleskov *et al*., 2019). It has been shown that water uptake was impaired and drought‐induced mortality of Norway spruce was amplified by high N deposition and subsequent nutrient imbalances (Schulze, 1989; Tresch *et al*., 2023). Both balanced fertilisation and N deposition reduce carbon (C)‐investment belowground (affecting both root and mycorrhizas) and thus increase drought susceptibility due to a decreased root‐to‐shoot ratio. It should also be noted that fertiliser application results in distinct and persistent changes to soil bacterial and fungal communities over extended time periods (Addison *et al*., 2019).

#### Ways to attenuate the physiological damage mechanisms

Nutrient imbalances – often caused by atmospheric deposition of reactive N – are known to lead to mortality, and drought exacerbates these nutrient imbalances (Kreuzwieser & Gessler, 2010). Given that balanced fertilisation with macro‐ and micronutrients, meant to avoid nutrient imbalances, can also increase drought stress (Ward *et al*., 2015), it seems in most cases inappropriate to intensively fertilise forest stands exposed to drought at present or in future. Gessler *et al*. (2017) provided a conceptual framework for analysing the interaction between drought and nutrients that might be used as scientific guidance for fertilisation: long‐term high nutrient availability (in forests that are not regularly drought‐exposed) is assumed to play a detrimental role in drought survival due to preferential biomass allocation aboveground that (1) predisposes plants to hydraulic constraints limiting photosynthesis and promoting hydraulic failure; (2) increases carbon costs during periods of carbon starvation; and (3) promotes biotic attack due to low tissue C : N. This predisposition might be partially compensated by higher nutrient availability in fertilised stands during a drought event by increasing the water use efficiency and minimising negative feedback between carbon and nutrient balance, as also observed by Schönbeck *et al*. (2020) for moderate drought. Thus, balanced and moderate fertilisation during longer‐term, low‐intensity drought might be an option to maintain root functioning (see Schönbeck *et al*., 2020) and fertiliser application after drought might facilitate tree recovery (Gessler *et al*., 2017). These findings are in agreement with other studies that have found partial compensatory effects of fertilisation on drought resistance (Ibáñez *et al*., 2018). Still, the overall effects of nutrient addition, especially in normally nondrought‐exposed forests, have a negative long‐term impact on resistance to drought events, leading to increased tree damage and mortality. Even though targeted fertilisation to mitigate nutrient restriction during drought or in the recovery phase is theoretically an option, trees are expected to show low nutrient uptake under these conditions (Gilliam, 2006). This may result in unwanted leaching of nutrients to the groundwater with the first precipitation events. General adverse effects of nutrient input in forest ecosystems that are normally N‐limited must be considered: High nutrient input and eutrophication jeopardise biodiversity (Nordin *et al*., 2005; Gilliam, 2006), which is, on the one hand, an important ecosystem service and, on the other hand, the basis for ecosystem functioning.

#### Further implications for climate‐smart forestry

Liming has been proposed as a means to mitigate the impacts of low pH and nutrient imbalances. However, the results are ambiguous, with often no or even adverse effects on tree growth and functioning (see review by Binkley & Hogberg, 2016). Only one study assessed the effect of liming on drought resistance, recovery and resilience of trees, hypothesising that liming may increase drought resistance owing to improved fine‐root growth (Kohler *et al*., 2019). The authors observed no difference between limed and unlimed plots in mitigating the impact of drought on Norway spruce during the event, and thus, no improved resistance upon liming. However, radial growth recovery and resilience after severe drought events were better in the liming treatments, indicating a shorter stress period. Even though the underlying mechanisms are unclear, liming might reduce spruce's susceptibility to secondary, drought‐related pests and pathogens. Given the limited knowledge on the impact of forest liming on drought resistance and resilience, further research is needed before evidence‐based advice can be provided to forest practitioners. We have: (1) to understand the mechanistic basis of liming on drought recovery and resilience; (2) to explore whether the results with spruce can be transferred to other tree species; and (3) to assess how different soil properties modify the liming effects.

As Fig. 5 shows, tree species mixtures can increase the belowground resource space, especially if soil nutrient resources are limiting. Thus, mixing tree species with complementary belowground resource acquisition strategies – rather than nutrient addition – may allow optimised stand nutrient supply, leading to increased ecosystem resistance and resilience to extreme events.

##### Key points for climate‐smart forest management

Fertilisation often increases the negative impact of drought on trees and forests. Although targeted fertilisation during drought could have potential beneficial effects, the overall negative consequences on biodiversity and ecosystem functioning must be considered. Liming may positively affect recovery and resilience, but systematic research is required before its application on a larger scale. Mixing tree species with complementary nutrient acquisition strategies may be the most sustainable way to optimise a forest ecosystem's nutrient supply without jeopardising its drought resistance and resilience.

## IV. Dryland mechanisms as targets of CSF management practices

Dryland mechanisms are individual adaptations and ecological processes that facilitate or assist organisms and, collectively, entire ecosystems to function in water‐limited environments (see Box 2; Fig. 2). While these mechanisms are primarily recognised in arid regions, some have been observed to support trees in humid forests experiencing drought (Grünzweig *et al*., 2022). Integrating dryland mechanisms in CSF may contribute to the resistance and resilience of selected trees and whole stands to extreme heat and drought, which are expected to occur more frequently in the temperate and boreal regions in the future.

Several key mechanisms that evolved in dryland ecosystems allow trees to acquire more water or use it more efficiently, despite low precipitation and high evaporative demand. Hydraulic redistribution (e.g. hydraulic lift from a deep to a shallow soil layer) is passive water movement along a gradient of water potential, from moist to drier soil through the root system (Neumann & Cardon, 2012). Water is commonly released from fine roots at night and gets reabsorbed by them during the day. In temperate and subtropical forests that are regularly exposed to dry periods, *c*. one‐third of soil water use and *c*. 40% of the water transpired by large trees were redistributed hydraulically (Brooks *et al*., 2002; Belovitch *et al*., 2024). In a rain exclusion experiment, mature beech redistributed water from moist to dry soil layers at a volume equivalent to 10% of its total transpired water (Hafner *et al*., 2025). In addition, forest regeneration benefited as tree seedlings of broadleaved and conifer species were able to absorb water redistributed by mature trees, such as *F. sylvatica* and *A. alba* in a central European forest during a dry period. Hence, hydraulic redistribution enhances resistance to drought in humid forests by supplying a water subsidy to an otherwise dry soil layer. This process supports root hydration and prevents root hydraulic failure of trees, reduces drought stress in associated mycorrhizas and in neighbouring plants, and may improve plant nutrition (Bachofen *et al*., 2024). Many trees world‐wide have shown the ability to hydraulically redistribute water (Bachofen *et al*., 2024), which offers an option to target major plant traits to enhance hydraulic redistribution when selecting tree species for mixed forests (see Section III.2). Such traits may include high root hydraulic conductivity, dimorphic rooting systems (combining deep and shallow roots), large xylem conduits, efficient soil–root contact, and low shoot capacitance (shoot water storage; Neumann & Cardon, 2012; Prieto *et al*., 2012; Quijano & Kumar, 2015; Sha *et al*., 2024).

Many tree species can increase their water reserves and enhance cell turgor and metabolic functions by taking up water through their leaves and bark (Berry *et al*., 2019). Tree canopies of species from many major families and genera, also in humid forests, were shown to directly absorb rainwater and humidity from atmospheric nonrainfall sources, such as fog and dew (Dawson & Goldsmith, 2018; Schreel & Steppe, 2020). Foliar water uptake during dry seasons enhanced the resistance of trees in tropical forests by supplying substantial amounts of water to leaves and significantly contributing to transpiration and photosynthesis of trees (Eller *et al*., 2013; Binks *et al*., 2019). Pathways of foliar water uptake include diffusion across the cuticle, absorption by stomata and trichomes, and entry through hydathodes (Berry *et al*., 2019; Schreel & Steppe, 2020). Consequently, the following leaf traits, which could be targeted by CSF, promote foliar water uptake: cuticle hydrophilicity and leaf pubescence (both contributing to surface wettability and water retention), cuticle permeability, stomatal uptake ability contributed to by stomata structure, leaf geometry (e.g. angle and curvature), and leaf thickness (Fernandez *et al*., 2017; Dawson & Goldsmith, 2018; Berry *et al*., 2019). Bark water uptake depends on anatomical and structural traits, such as thin bark, high permeability, low suberization, and the presence of surface fissures or lenticels (Van Stan *et al*., 2021), and multitrait foliar ‘uptake syndromes’ have been defined for trees (Chin *et al*., 2023). In a changing climate, the ability to absorb both small amounts of rainwater intercepted by the canopy and nonrainfall humidity (mainly dew and water vapour) may become important sources of moisture (Schreel & Steppe, 2020). Moreover, pubescent leaves may even enhance dew condensation and prolong wetness, while also contributing directly to water absorption (Konrad *et al*., 2014). Screening for native and non‐native forest tree species, which efficiently exploit nonsoil water resources, might allow the inclusion of such species in the CSF species portfolio at sites where fog is assumed to occur often, also under future climate scenarios.

Water absorbed through the canopy can be transported inside the plant, even reaching the roots, through the reverse flow mechanisms of hydraulic redistribution (Eller *et al*., 2013; Schreel & Steppe, 2020). This suggests that foliar water uptake and hydraulic redistribution may function in tandem to buffer trees against water deficits across above‐ and belowground compartments. The selection criteria for species and provenances to meet CSF goals could, hence, include the ability of foliar and bark water uptake together with efficient hydraulic redistribution to improve tree water status and performance under extreme hot and dry conditions. If planted in diverse forest stands, such species might provide strong facilitation effects for the whole stand.

Beyond the individual level, the canopy convector effect at the stand level enhances air‐cooling of trees by sensible heat flux when evaporative cooling via transpiration is constrained by the lack of water. This dryland mechanism arises from the physical roughness of the forest canopy, which depends on the structural heterogeneity reducing the aerodynamic resistance and enhancing turbulent exchange of heat between the canopy and the atmosphere (Rotenberg & Yakir, 2010; Banerjee *et al*., 2017). Under strong surface heating, the canopy convector effect intensifies and becomes particularly efficient. During extreme heatwaves in Europe, it enabled forests to cool efficiently by sensible heat, while using water conservatively (Teuling *et al*., 2010). This cooling effect is particularly important given that heat stress can compromise leaf cuticular barriers and increase nonstomatal water loss (see Section II.1), making canopy‐level temperature regulation a critical component of forest drought resilience. CSF can aim to enhance the canopy convector effect by mixing selected tree species and specifically creating structurally (i.e. horizontally and vertically) diverse, multi‐aged stands at reduced density, in line with the measures discussed in Sections III.3 and III.4.

Overall, the framework of CSF can contribute to the heat and drought resistance and the resilience of the forest by explicitly targeting the operation of dryland mechanisms at both the tree and stand levels. The silvicultural measures needed are compatible with those of the more ‘traditional’ CSF methods described in Section III, even though the trait selection for trees in mixtures might be more specific in order to target, for example, foliar water uptake and hydraulic redistribution. More research is needed to better understand which specific combination of dryland mechanisms CSF should target, which ones can be best linked with the CSF measures in Section III, and which ones could either act as preventive measures or should be included in reactive management options instead. This latter distinction is essential as timber production or other ecosystem services, as well as the competitiveness of particular species, could be negatively impacted under nondrought conditions.

## V. Conclusions

Climate change is rapidly altering the boundary conditions for forestry. CSF is meant as a strategy to address these challenges and adapt forests to mitigate the effects of climate change, while supporting biodiversity and providing multiple, balanced ecosystem services. CSF measures can be directly targeted to mitigate direct and indirect drought effects via three pathways:

1. Reducing the water demand of the stand through thinning. However, practice needs to account for the long‐term environmental context to avoid adverse effects related to overbuilt crowns.
2. Belowground niche complementarity in mixtures can lead to a more efficient use of the available water resources and thus avoid negative drought impacts. Complementarity may be combined with facilitation effects by mixing in species that provide foliar water uptake and hydraulic redistribution. However, as with thinning, the long‐term conditions need to be accounted for. Tree diversity experiments, such as those in the network TreeDivNet (https://treedivnet.ugent.be/?cmdf=treedivnet, accessed on 20 July 2025), will, in the long term, provide a better understanding of the mechanisms underlying the positive or negative effects of diversity on drought resistance, resilience, and recovery.
3. Structural diversity and different age classes may increase the resource complementarity effects of species mixtures and monocultures. Reducing tree height via shorter production times and changing intervention regimes can directly decrease the hydraulic vulnerability of trees. However, we need more information on how such measures affect below‐ and aboveground trait coordination, and whether, for example, a reduced root‐to‐shoot ratio counteracts the positive effects of reduced tree height. In addition, the potential negative impacts of shorter production times on biodiversity and ecosystem services must be considered (Jandl *et al*., 2024).

Overall, a combination of different measures (uneven‐aged mixed forests with targeted thinning treatments and shorter production times) is likely to provide the best results for adjusting forests to the expected heat and drought effects, and we should be open to learning from dryland forests and implementing dryland mechanisms in CSF schemes. While tree diversity experiments that assess the impact of species and functional diversity (e.g. TreeDivNet) and CSF trials (https://forwards‐project.eu/eu‐csf‐network/, accessed on 21 January 2026) are being established, there is no concerted effort yet to bring these experiments and networks together. The current lack of interaction may be due to the fact that further trials are primarily led by ecologists, while the latter are managed by foresters. We need, however, joint trials and experiments to better understand and predict how diversity‐ and management‐related factors concertedly affect the physiology of drought resistance and resilience. Since trees are long‐lived organisms, this requires considerable staying power, but given the threats climate change poses to our forests, it should be a priority and deserves world‐wide attention and coordination.

## Competing interests

None declared.

## Disclaimer

The New Phytologist Foundation remains neutral with regard to jurisdictional claims in maps and in any institutional affiliations.

## Supporting information


**Fig. S1** Present potential distribution of 38 native forest tree species in Switzerland and changes to be expected in the future as calculated by an ensemble of species distribution models.
**Table S1** The 10 most abundant tree species as shown in Fig. 4.
Please note: Wiley is not responsible for the content or functionality of any Supporting Information supplied by the authors. Any queries (other than missing material) should be directed to the *New Phytologist* Central Office.
