# Supplementary material for: Shaping future forests: how can ecophysiology support climate‐smart forest management?
Source: New Phytol. 2026 Feb 23;250(5):2778–813. doi: 10.1111/nph.71007 (PMC13150318; doi:10.1111/nph.71007)
Supplement: Supplementary file 1 — Fig. S1 Present potential distribution of 38 native forest tree species in Switzerland and changes to be expected in the future as calculated by an ensemble of species distribution models. Table S1 The 10 most abundant tree species as shown in Fig. 4. Please note: Wiley is not responsible for the content or functionality of any Supporting Information supplied by the authors. Any queries (other than missing material) should be directed to the New Phytologist Central Office. [file NPH-250-2778-s001.pdf]

New Phytologist **Supporting Information**

Article title: ***Shaping Future Forests: How can ecophysiology support climate-smart forest management?***

Authors: *Arthur Gessler, José M Grünzweig, Laura Bigio, Henrik Hartmann, Nate McDowell, Frank Krumm, Arun K Bose, Andreas Rigling, Harald Bugmann, Valentina Vitali, Pascal Schneider, J Jelle Lever, Janine Schweier, Anne Kempel, Niklaus E Zimmermann, Philipp Brun, Jürgen Bauhus, Micah Wilhelm, Alessandra Bottero*

Article acceptance date: 17 January 2026

[Correction added on 9 March 2026, after first online publication: panels in Fig. S1 have been updated.]

The following Supporting Information is available for this article:

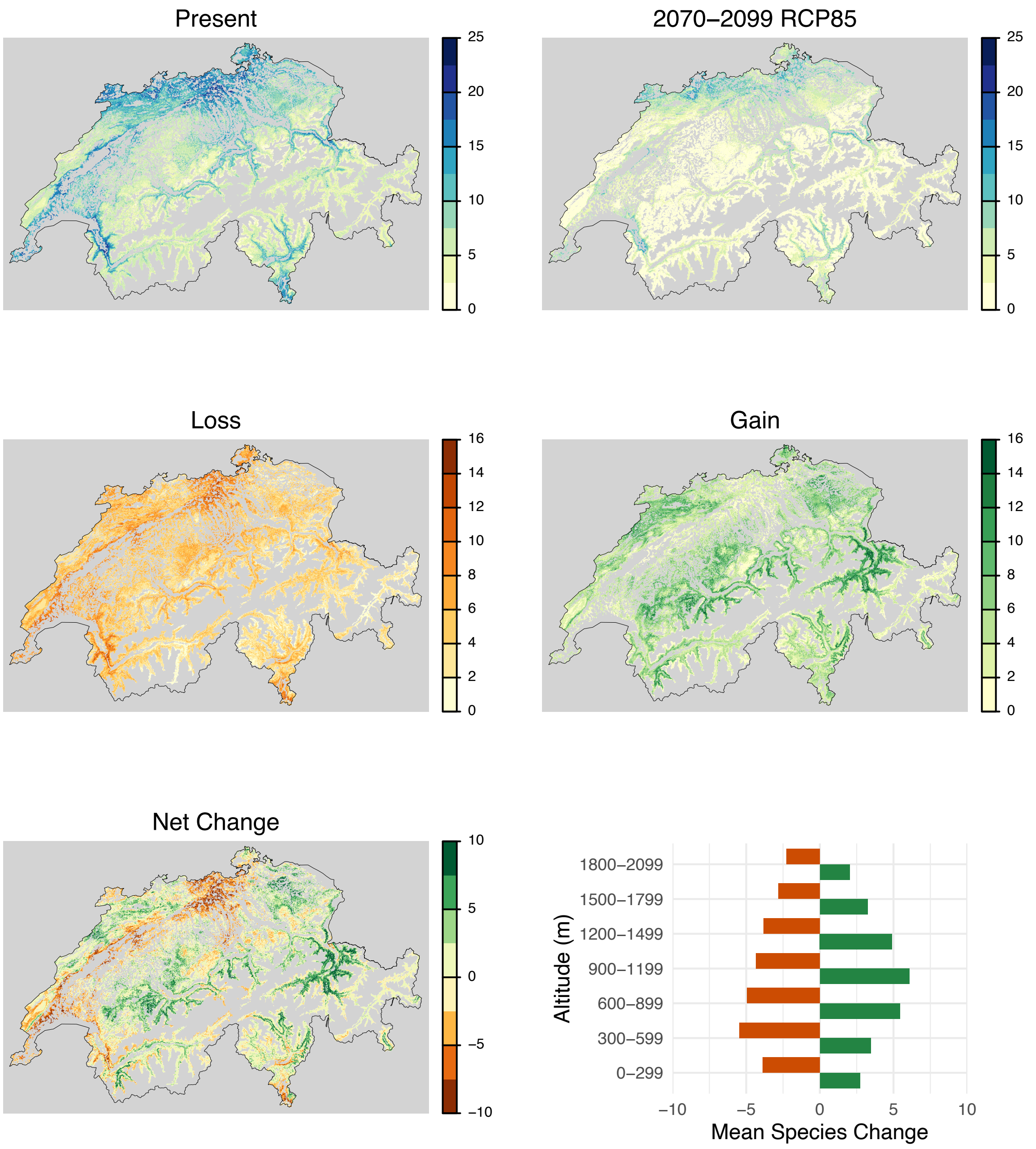

Fig. S1 Present potential distribution of 40 native forest tree species in Switzerland and changes to be expected in the future as calculated by an ensemble of species distribution models. The different panels show the number of species that have potentially suitable conditions at a given pixel under present climatic conditions, the number of species suitable under today's climate and

by the end of the century, assuming the RCP 8.5 scenario, the gross loss, gross gain, and net change in the number of species, respectively. The panel at the lower left side depicts the altitudinal distribution of losses and gains in the number of species. The colour codes indicate the number of species. The following species have been included:

Abies alba, Acer campestre, Aceropalus, Acer platanoides, Acer pseudoplatanus, Alnus glutinosa, Alnus incana, Betula pendula, Betula pubescens, Carpinus betulus, Castanea sativa, Fagus sylvatica, Fraxinus excelsior, Fraxinus ornus, Juniperus communis, Laburnum anagyroides, Larix decidua, Ostrya carpinifolia, Picea abies, Pinus cembra, Pinus mugo, Pinus nigra, Pinus strobus, Pinus sylvestris, Populus tremula, Prunus avium, Prunus padus, Quercus cerris, Quercus petraea, Quercus pubescens, Quercus robur, Salix alba, Sorbus aria, Sorbus aucuparia, Sorbus torminalis, Taxus baccata, Tilia cordata, Tilia platyphyllos, Ulmus glabra, Ulmus minor

Table S1 The 10 most abundant tree species as shown in Figure 4

Picea abies

Abies alba

Fagus sylvatica

Pinus sylvestris

Larix decidua

Quercus robur

Quercus petraea

Acer pseudoplatanus

Alnus glutinosa

Fraxinus excelsior
